# Optimization of the standard genetic code according to three codon positions using an evolutionary algorithm

**DOI:** 10.1371/journal.pone.0201715

**Published:** 2018-08-09

**Authors:** Paweł Błażej, Małgorzata Wnętrzak, Dorota Mackiewicz, Paweł Mackiewicz

**Affiliations:** Department of Genomics, Faculty of Biotechnology, University of Wrocław, Wrocław, Poland; UMR-S1134, INSERM, Université Paris Diderot, INTS, FRANCE

## Abstract

Many biological systems are typically examined from the point of view of adaptation to certain conditions or requirements. One such system is the standard genetic code (SGC), which generally minimizes the cost of amino acid replacements resulting from mutations or mistranslations. However, no full consensus has been reached on the factors that caused the evolution of this feature. One of the hypotheses suggests that code optimality was directly selected as an advantage to preserve information about encoded proteins. An important feature that should be considered when studying the SGC is the different roles of the three codon positions. Therefore, we investigated the robustness of this code regarding the cost of amino acid replacements resulting from substitutions in these positions separately and the sum of these costs. We applied a modified evolutionary algorithm and included four models of the genetic code assuming various restrictions on its structure. The SGC was compared both with the codes that minimize the objective function and those that maximize it. This approach allowed us to place the SGC in the global space of possible codes, which is a more appropriate and unbiased comparison than that with randomly generated codes because they are characterized by relatively uniform amino acid assignments to codons. The SGC appeared to be well optimized at the global scale, but its individual positions were not fully optimized because there were codes that were optimized for only one codon position and simultaneously outperformed the SGC at the other positions. We also found that different code structures may lead to the same optimality and that random codes can show a tendency to minimize costs under some of the genetic code models. Our results suggest that the optimality of SGC could be a by-product of other processes.

## Introduction

The standard genetic code (SGC) is one of the most intriguing products of evolution. Its origin and uniqueness remain mysterious, especially if we take into account the extremely large number of possible alternatives built from 61 codons encoding 20 amino acids and three stop translation codons. This figure is around 1.51⋅10^84^ [1], which substantially exceeds the number of hydrogen atoms in our observable universe. The large number of possible codes suggests that the standard genetic code can be a “frozen accident” that was randomly selected from so many possibilities and then fixed because any further changes had disastrous consequences for the large number of already synthetized proteins [2].

On the other hand, even before the final deciphering of all codons in the SGC [3, 4], many assignments of amino acids with specific physicochemical properties to similar codons were noted, which implied that the structure of the standard genetic code could have evolved to minimize the lethal effects of mutations [5] and errors occurring during protein synthesis [6]. Thereby, an adaptive hypothesis about SGC evolution was put forward. It was tested by other researchers, who demonstrated a tendency of the genetic code to minimize such errors according to the physicochemical properties of amino acids (e.g. polarity) and folding free energy of protein structures [7–20].

However, this tendency of the SGC was not supported by all analyses. Novozhilov, Wolf [21] concluded from their study that the SGC is only partially optimized for robustness to translational errors. Higgs [22] proposed that minimization of the translational error was not the main force that drove genetic code evolution but rather the addition of subsequent amino acids into positions previously occupied by other amino acids with similar physiochemical properties decided the final structure of the code. Moreover, extensive searches of the space of alternative codes without restrictions on their structure showed that the SGC is far from the optimum and that it is possible to find optimal genetic codes that minimize the costs of amino acid replacements much better than the standard genetic code [23, 24].

It has also been postulated that the properties of the SGC to minimize the costs of amino acid replacements evolved as a by-product of the genetic code expansion associated with the duplication of genes encoding adaptor molecules and charging enzymes [25, 26]. Under this model, no directional selection was necessary to create such features of the SGC [27].

The role of error minimization in SGC evolution was also diminished by the coevolution hypothesis, which assumes that the assignment of amino acids to their codons proceeded according to their relationships in biosynthetic pathways [28–36]. An amino acid that was synthesized from a precursor took over some of its codons in the genetic code. An alternative proposition called the stereochemical hypothesis assumes that some interactions between amino acids and nucleotide aptamers played a central role in the early stages of SGC evolution [37–41]. According to the Coding Coenzyme Handle Hypothesis (CCH), such interactions between amino acids and specific oligonucleotides called handles could have improved the catalytic effectiveness of ribozymes in the RNA world because of the chemical diversity of amino acids [42, 43]. Thereby, amino acids played a role of coenzymes or cofactors for the ribozymes. The handles with linked amino acids could attach to specific sites of ribozymes via base pairing with the cognate triplet of the handles. Later, the handles evolved into adaptors (tRNA), ribozymes were replaced by proteinous aminoacyl-tRNA synthetases, and ribozymes became mRNAs losing their original enzymatic activity. In this concept, the ribozyme’s sites attached to the handle’s triplets would correspond to codons, while the triplets to anticodons.

However, these concepts have also been criticized. The relationship between precursor–product pairs of amino acids and the codon similarity turned out statistically insignificant, while some precursors and products proved to be charged by tRNAs with different core group structures [27, 44–46]. Critics of the stereochemical hypothesis emphasize that many interactions between aptamers and amino acids are weak and nonspecific or unobserved at all [47, 48]. Only very few cases of the interactions were strong or statistically significant and usually involved more complex amino acids that are believed to be late additions to the genetic code. However, simpler amino acids and those identified in meteorites and produced in prebiotic chemistry experiments should be expected in this case. The CCH is also poorly supported by experiments because the catalytic help of ribozymes by amino acids was showed only for histidine [49, 50]. Nevertheless, the proposed hypotheses are not mutually exclusive and may describe different stages of the genetic code evolution.

Since there is no consensus about the origin of the SGC, this subject is worthy of further investigation. Regardless of the question as to whether the SGC is globally optimal or not, it is interesting to study SGC optimality in terms of its individual features because it may help us identify the factors influencing the origin and evolution of the standard genetic code.

An important feature that should be taken into account in studying the SGC optimality is the different impacts of mutations in individual codon positions. It is well known that the second codon position is the most conserved position because every nucleotide change in this position results in an amino acid replacement [6, 7, 51]. The most deleterious mutations are substitutions between adenine and thymine because these mutations cause changes in the polarity and hydropathy of coded amino acids. Five synonymous substitutions are possible in the first codon position, whereas the most degenerated position is the third one. This position includes 13 two-fold degenerated sites in which two nucleotides specify the same amino acid, one three-fold degenerated site in which changes between three nucleotides have no effect on the encoded amino acid, and eight four-fold degenerated sites, which tolerate all possible nucleotide mutations. A nucleotide in the third codon position of mRNA can also form nonconventional (wobble) base-paring with a nucleotide in the first position of a tRNA anticodon [52]. The codon positions can also differ by the type and level of translational errors that they are subjected to [53–56]. Although some weights for these errors were proposed [9], they were criticized because the experimental data were not consistent and their results could not be generalized (see the comments by David Ardell on the paper by Novozhilov, Wolf [21]).

Taking into account the different features of the three codon positions, we focused on the problem of optimality of the standard genetic code by considering the costs of amino acid replacements resulting from substitutions in these positions separately and the sum of these costs. To find the optimal codes, we applied a specific version of an evolutionary algorithm (EA) [57], which seems to be a better approach in the study of genetic code optimization than the classic comparison of the SGC with randomly generated theoretical alternatives [7–10, 58] due to the large number of possible alternative codes and extremely vast search space. The random codes represent only a very tiny fraction of all possibilities and are not necessarily representative of the whole space of the theoretical codes. The EA technique was already successfully applied in various studies on the optimality of the genetic code [23, 24, 59–62].

Furthermore, in contrast to previous analyses, we searched not only for the codes that minimize the cost function but also for codes that maximize it. Such an approach enabled us to place the SGC in the space of possible codes more accurately than has been done in analyses based on randomly generated codes. Moreover, we analysed the influence of the optimization of one codon position on the optimality of other non-optimized positions in order to study the relationships between the codon positions in terms of genetic code optimization. Moreover, we investigated the optimized codes under four models of the genetic code assuming various restrictions on its structure. The proposed approach allowed us to assess the importance of individual codon positions for the optimality of the standard genetic code in the context of its structure.

## Methods

### Evolutionary algorithm

To search for the optimal genetic codes, we applied an evolutionary algorithm (EA) technique [57]. In this method, the hypothetical genetic codes are treated as individuals, creating a population of potential solutions. The initial population consists of randomly generated codes, which are then modified in the processes of mutation and crossover. Both processes generate new individuals in the population. The modified population is subjected to the selection process in which the fitter individuals, regarding the criteria of optimality, pass with a high probability to the next simulation step. The generation of variability and the selection process are alternately repeated throughout the whole simulation until the algorithm attains parameter stability.

### Models of genetic codes and genetic operators

The mutation and crossover processes are realized in the EA by appropriate genetic operators. In our case, their representation depends on the considered models of the genetic code. We studied the optimization of the SGC under four models (codon assignment methods) with various levels of restrictions imposed on the genetic code structure:

1. The DEG (codon degeneracy) model preserves the degeneracy and codon block structure, i.e., groups of codons, characteristic of the SGC. The potential codes were generated by permutation of the amino acid assignments between codon blocks with the same degeneracy, which means that only the blocks with the same number of codons could exchange their amino acid assignments;
2. The BLO (codon blocks) model preserves the codon block structure characteristic of the SGC. The potential genetic codes were generated by permutation of the amino acid assignments between codon blocks, disregarding their degeneracy (e.g. a block consisting of two codons could exchange its amino acid assignment for an amino acid encoded by a block of four codons);
3. The NUM model (codon number) preserves the number of codons per amino acid as in the SGC. The potential genetic codes were generated by random assignments of amino acids to various codons but the number of codons for a given amino acid was maintained as in the SGC; and
4. The GEN model (general case) has no constraints on the genetic code structure and assumes only that every amino acid should be coded by at least one codon. The potential genetic codes were generated by a random division of 61 codons into 20 amino acids.

All the models assumed the presence of all 20 amino acids and the stop translation signal in every individual, which is the same as in the SGC. Additionally, in all these models we considered only the codes with exactly the same three stop translation codons as in the SGC. Mutations were accomplished by random exchange of codon blocks (in the cases of the DEG and BLO models) or codons (in the NUM and GEN models) between two selected amino acids. The crossover operators were also adjusted to models of the genetic code.

### Crossover operators used in the evolutionary algorithm

The crossover operator was properly adjusted to the models of the genetic code. In the case of DEG and BLO models, we modified the Position Based Crossover (POS) operator [63], which is often used in evolutionary-based approaches to the travelling salesman problem. This operator selects amino acids randomly from parental codes (*P*_1_ and *P*_2_) and assigns them to the corresponding codon blocks in their corresponding offspring (*O*_1_ and *O*_2_), i.e. *P*_1_→*O*_1_ and *P*_2_→*O*_2_. The remaining codon blocks in the offspring have assigned amino acids from other parent, i.e. *P*_1_→*O*_2_ and *P*_2_→*O*_1_. The amino acids are assigned according to the amino acid order in the parental code. When an amino acid is already present in the offspring, the other one is selected according to its position in the set of amino acids. Thanks to that every amino acid in the offspring is assigned only to one codon block. In the DEG model, amino acids were interchanged only between codon blocks with the same degeneracy, whereas in the BLO model, the swap between codon blocks with different degeneracy was also allowed.

However, the POS procedure cannot be applied in the cases of NUM and GEN models because it could cause the loss of some of the amino acids from the genetic code. Therefore, we developed another version of the crossover operator, which is fully described in [23]. At the beginning, two offspring *O*_1_ and *O*_2_, identical to their corresponding parents *P*_1_ and *P*_2_, are created. Next, an amino acid, e.g. *a*_1_, the same in the two parents, is randomly selected. If the same codons of this amino acid exist in two parental codes, no exchange of codons is performed in offspring. If codons for this amino acid are present in one parent and absent in the other, we select the pair of such codons and exchange them mutually in two offspring. If there are no codons for the amino acid *a*_1_ in one parent to swap but the second parent *P*_2_ has still a codon for this amino acid, we move this codon form other amino acid, e.g. *a*_2_, and assign to *a*_1_ in the offspring *O*_1_. Simultaneously, in the offspring *O*_2_, this codon is shifted from *a*_1_ to the other amino acid, e.g. *a*_3_. To ensure that each amino acid is coded by at least one codon, codons that are the only ones for the given amino acid are not moved. In the NUM model, the swap of codons was carried out to maintain the number of codons for the given amino acid as in the SGC, whereas this condition was not taken into account in the most general GEN model.

### Objective function

The optimization level of genetic codes in the evolving population was assessed by an objective function, which is the sum of all squared differences between the polarity values of amino acids encoded by codons before and after a single-point mutation:

$$F_{}=\sum_{<i,j>\in D}[p(i)-p(j){]}^{2},$$ (1)

where *D* is the set of pairs of codons <*i*,*j*>, which differ in one codon position, whereas *p(i)* and *p(j)* are the polarity values of amino acids coded by the codons *i* and *j*, respectively. The values of polarity were taken from the amino acid index presented by Woese [64]. A low value of the function *F* means better minimization of the effects of amino acid replacements by a genetic code.

This type of fitness measure is commonly used in estimation of the quality of genetic codes in many approaches [7, 9, 20, 24, 60, 61] because it appears to be a good physicochemical parameter describing an important property of proteins.

We considered two ways to optimize the objective function: minimization and maximization. The aim of the first one is to find the genetic codes that minimize the costs of mutations or mistranslations regarding polarity. The second approach refers to the opposite extreme in which the structure of the genetic code leads to the largest differences in the polarity of replaced amino acids. Aside from the total sum of differences between the polarity values of amino acids resulting from single mutations in all codon positions *F*_*T*_, we also optimized the objective function for substitutions in three codon positions (*F*_1_, *F*_2_ and *F*_3_) separately.

### Measures of genetic code optimality

To assess the optimality of the standard genetic code, we used a modified measure invented by Di Giulio [20]. The original measure, called percentage distance minimization (pdm), calculates the percentage in which the standard genetic code differs from the randomized mean code in relation to the distance between the randomized mean code and the best optimized one. However, the randomly generated subset of codes may not represent all the possible codes well and therefore may not be the best reference. Therefore, we proposed a new measure, called global distance, which compares the optimality of SGC with two extreme genetic codes, i.e., ones that minimize and ones that maximize the objective function:

$$GD=\frac{F^{SGC}-F^{best}}{F^{worst}-F^{best}}\cdot 100\%,$$ (2)

where *F*^SGC^ is the objective function value of the standard genetic code, *F*^best^ is the objective function value obtained for the best found alternative that minimizes the polarity costs and *F*^worst^ is the objective function value calculated for the worst found alternative code that maximizes the polarity costs. The main advantage of the *GD* is the ability to present the level of optimization of the SGC in a scale that takes into account the two extreme optimization cases and thus approximates the whole space of possible genetic codes. Such a distance was calculated in cases where we optimized the polarity costs for three codon positions individually *GD*_1_,*GD*_2_,*GD*_3_ and the sum of polarity costs for all three positions *GD*_T_. Replacing *F*^SGC^ by the mean objective function value for the randomized (starting) codes *F*^rand^ in Eq (3), we also calculated the normalized distance between the average randomized code and the best code by minimizing the polarity costs $GD_{1}^{rand},GD_{2}^{rand},GD_{3}^{rand},GD_{T}^{rand}$.

To determine the location of the standard genetic code in the three-dimensional space of three objective function values for three codon positions, i.e., *F*_1_,*F*_2_,*F*_3_, we also calculated the shortest Euclidean distances between the SGC and the best optimized codes *ED*^best^ as well as the worst optimized codes *ED*^worst^:

$$ED^{\mathrm{best}}=\sqrt{{\left(F_{1}^{\mathrm{SGC}}-F_{1}^{\mathrm{best}}\right)}^{2}+{\left(F_{2}^{\mathrm{SGC}}-F_{2}^{\mathrm{best}}\right)}^{2}+{\left(F_{3}^{\mathrm{SGC}}-F_{3}^{\mathrm{best}}\right)}^{2}},$$ (3)

$$ED^{\mathrm{worst}}=\sqrt{{\left(F_{1}^{\mathrm{SGC}}-F_{1}^{\mathrm{worst}}\right)}^{2}+{\left(F_{2}^{\mathrm{SGC}}-F_{2}^{\mathrm{worst}}\right)}^{2}+{\left(F_{3}^{\mathrm{SGC}}-F_{3}^{\mathrm{worst}}\right)}^{2}}.$$ (4)

The analogous distances were calculated for each random (starting) genetic code, replacing *F*^SGC^ by the objective function value of a randomized code *F*^rand^. Finally, the average distances for all randomized codes, $\bar{ED^{\mathrm{best}}}$ and $\bar{ED^{\mathrm{worst}}}$, were obtained.

### Comparison of the genetic code structure

To assess the differences between the structures of the genetic codes, we considered all 1220 possible assignments of 20 amino acids into 61 codons (three stop codons were fixed as in the SGC) and marked the presence (1) or absence (0) of the given assignment for individual codes. Next, we calculated the Hamming distance between two codes represented by such binary strings, and we normalized it by 122, i.e., the maximum possible distance that can be obtained for the codes with 61 unfixed codons. We called this measure the structure distance *SD*, and it is given by the following formula:

$$SD=100\cdot \sum_{i=1}^{1220}d_{i}^{a,b}/122,$$ (5)

where *i* is the assignment of an amino acid to a codon; if the assignment *i* is the same in the two compared genetic codes *a* and *b*, then $d_{i}^{a,b}=0$; if the assignment *i* is different in the two compared genetic codes, then $d_{i}^{a,b}=1$. The maximum distance *SD* that can be obtained is 100%, and the minimum is 0%. Moreover, the genetic codes presented in the form of binary strings were compared in the correspondence analysis for visualization purposes using the CA function from the R package FactoMineR [65].

### Simulation procedure

The simulations were run through 1000 generations and were repeated 20 times with different seeds. After testing various values of the genetic operators, we applied the mutation probability 0.9 and the crossover probability 0.3 for the DEG and BLO models, and the mutation probability 0.8 and the crossover probability 0.4 for the NUM and GEN models. The simulation started with 1000 random genetic codes for the simpler models (DEG and BLO) and with 2800 random genetic codes for the complex models (NUM and GEN). The evolving population of the genetic codes consisted of parent (regular) and archive (external) populations. After the selection of 700 best solutions from these sets, the archive set was updated and then subjected to the binary tournament. The winning individuals increased their diversity by mutations and crossover to become a new parent set.

Each simulation was checked for its stability and convergence. An example of the change in the objective function values during simulation time for the most complex model of the genetic code is shown in S1 Fig in Supporting Information.

## Results

### Optimality of the standard genetic code regarding three codon positions and four models of the genetic code

Using the EA technique, we assessed the optimality of the standard genetic code (SGC) regarding two aspects: the model of the genetic code and codon positions. We considered four models of the genetic code with different levels of restrictions on the structure (see Materials and Methods for details). The least restrictive model (the GEN model) assumed only the presence of 20 amino acids and 61 codons, whereas the others preserved some features of the SGC: the number of codons per amino acid (the NUM and DEG models) or the codon block structure (the BLO and DEG models). All the models maintained three stop translation codons as in the SGC. Consequently, the models differed in the complexity and assumed various numbers of possible codes: 8.788·10^78^ (the GEN model), 5.559·10^64^ (the NUM model), 2.433·10^18^ (the BLO model) and 5.225·10^8^ (the DEG model). The three codon positions play different roles in coding for protein sequences and their translation process; therefore, they were considered individually aside from the optimization of the SGC with respect to the total costs for all three codon positions.

**Fig 1:**
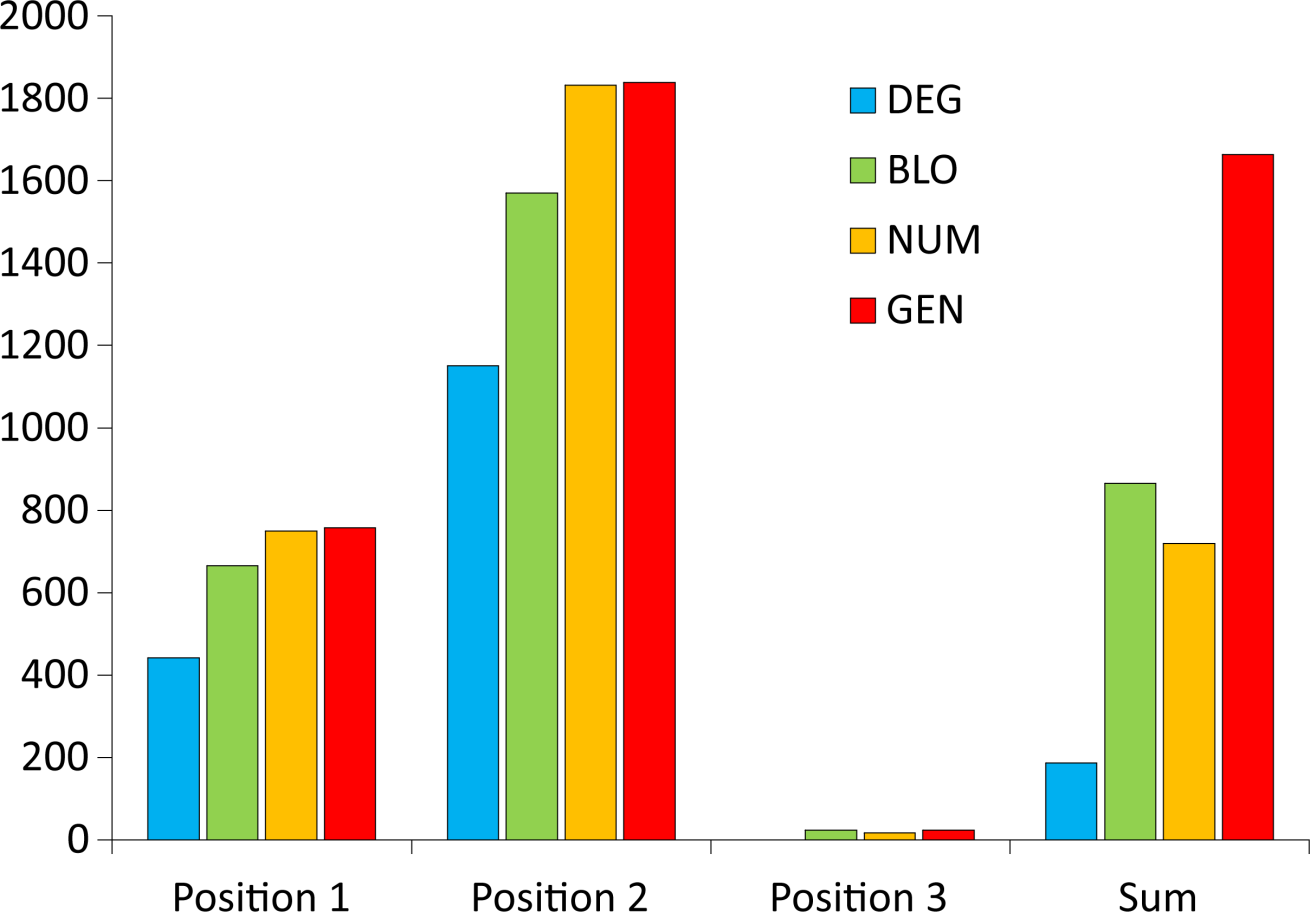
The difference between the values of objective function of the SGC (*F*^SGC^) and the best code (*F*^best^) calculated under four models with different restrictions on the genetic code structure when the polarity costs were minimized for the three codon positions individually or as the sum of costs over all these positions; GEN, the least restrictive model; NUM, the model preserving the number of codons per amino acid; BLO, the model preserving the codon block structure; DEG, the model preserving the degeneracy.

At first, we directly compared the objective function values of the SGC and the best code by calculating the difference between them, i.e., *F*^SGC^−*F*^best^, for various models of the genetic code and optimization criteria (Fig 1). Generally, the more complex the model, the greater the difference and the weaker the ability of the SGC to minimize the polarity costs. The most distinctive results are those of the optimizations regarding the third codon position, which have the smallest differences. This finding implies that the SGC minimizes the costs of amino acid replacements in this position similarly to the best codes, especially under the DEG model. On the other hand, the amino acid replacements resulting from the nucleotide substitutions in the second codon position are the least optimized in the SGC.

A popular assessment of genetic code performance is percentage distance minimization (pdm), which compares the SGC to a randomized mean code and the best optimized one. However, the simple difference between the polarity cost of the SGC and the best code is not a good measure because it does not take into account the whole space of possible cost values. Similarly, the comparison of the SGC with a subset of randomly generated codes is not a good parameter either because random sampling of genetic codes from such a large number of genetic code possibilities may be biased, method-dependent, or may not cover the whole space of possible costs. For example, the assignment of each of the 20 amino acids with equal probabilities to at least one of the 61 codons will preferably generate codes with nearly equal numbers of codons per amino acid.

**Fig 2:**
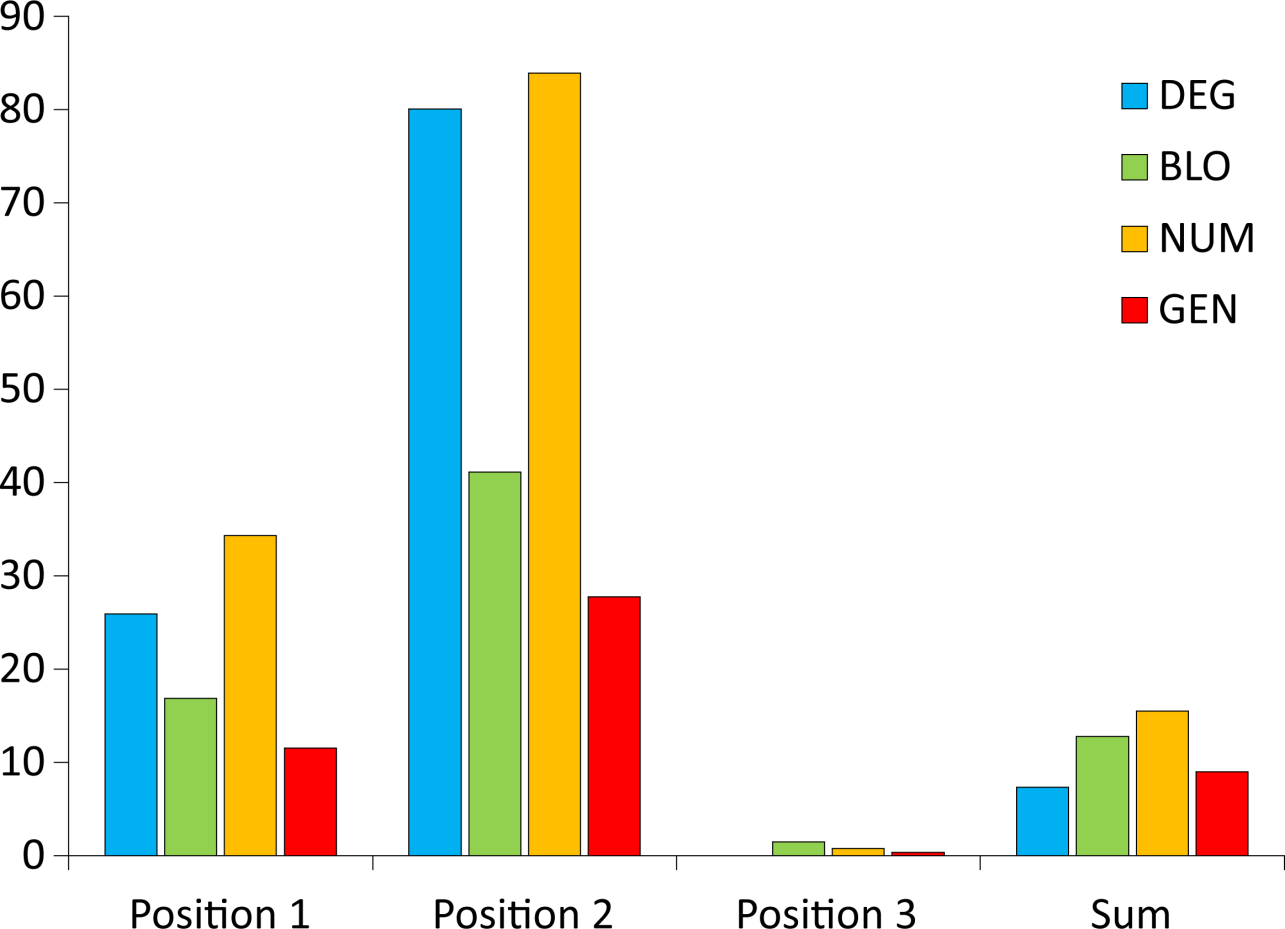
The *GD* measure calculated under four models with different restrictions on the genetic code structure when the polarity costs were minimized for three codon positions individually or as the sum of costs over all positions; GEN, the least restrictive model; NUM, the model preserving the number of codons per amino acid; BLO, the model preserving the codon block structure; DEG, the model preserving the degeneracy.

Therefore, to put the cost of the SGC in the whole space of costs of amino acid replacements, we compared the SGC not only with random codes and those that minimize these costs but also with codes that maximize them. To study these codes, we introduced a new measure called the global distance, *GD* (Eq 3), which compares the SGC with two types of codes that minimize and maximize the costs. The measure ranges from 0% to 100% and presents the optimization ability of the SGC in the global space. Small *GD* values indicate that the SGC has a tendency to minimize the costs, whereas large values imply that it maximizes them. *GD* equal to 50% means that the code optimality is equidistant from these two extremes. Fig 2 presents the *GD* measure obtained for the studied models of the genetic code and four optimization criteria.

Interestingly, the relationship between the complexity of the genetic code model and the *GD* optimality measure, which was visible for the simple difference between the objective function values of the SGC and the best code in Fig 1, disappears here (Fig 2). Nevertheless, the third codon position also shows the best level of polarity cost minimization, ranging from 1.5% (the BLO model) to 0% (the DEG model), whereas the second codon position is the worst optimized in this respect (Fig 2). The optimization level of this position is closer to the worst found code under the DEG and NUM models, i.e., 80.1% and 83.9%, respectively. In the BLO and GEN models, this position shows a tendency to minimize the costs at 41.1% and 27.7%, respectively, but it is still much less optimized than the other codon positions in the respective genetic code models. The first codon position, depending on the genetic code model, is optimized from 34.3% to 11.5%. Low *GD* values were found for the sum of the costs in three codon positions, ranging from 15.5% (the NUM model) to 7.3% (the DEG model). For the most general model (GEN), this value was 9.0%.

From the results presented above, we can draw some general conclusions concerning the optimality of the SGC. The first one is that the greater the number of restrictions on the genetic code structure imposed in the searched models, the smaller the difference between the values of the function *F* of the SGC and that of the best codes. However, even for the most restrictive model, it is possible to find a code that minimizes the amino acid replacement costs better than the SGC. The best minimized solutions were found among the codes under the least restricted model, which indicates that the structure of the SGC can be improved according to the assumed criteria. The second conclusion is that comparing the SGC with both the best and worst codes gave us a much more realistic placement of the SGC in the space of theoretical genetic codes. The SGC was well optimized for the sum of costs for all codon positions, reaching a general distance to the best codes of up to approximately 16% in the global space. The analysis of SGC optimality in the individual codon positions showed that the block structure of the SGC is almost perfectly optimized for the minimization of amino acid replacement costs for the third codon position, regardless of the genetic code model assumed. The SGC is least optimized for the second codon position, also regardless of the genetic code model. The optimization of this position is even much closer to the worst codes than the best ones for some of the models. Therefore, the SGC does not seem be optimized as well as the adaptive code hypothesis would claim.

### Influence of the optimization of one codon position on the optimality of other positions

The results presented above focused only on the costs optimized for individual codon positions or for all positions simultaneously. However, it is interesting to see what the polarity costs in the non-optimized codon positions are when the costs are optimized for only one other position or all three codon positions. Such a comparison can uncover important relationships between the codon positions in terms of genetic code optimization, for example, if it is possible to improve two codon positions simultaneously or one codon position without significant deterioration of the others. Therefore, we explored this subject.

**Fig 3:**
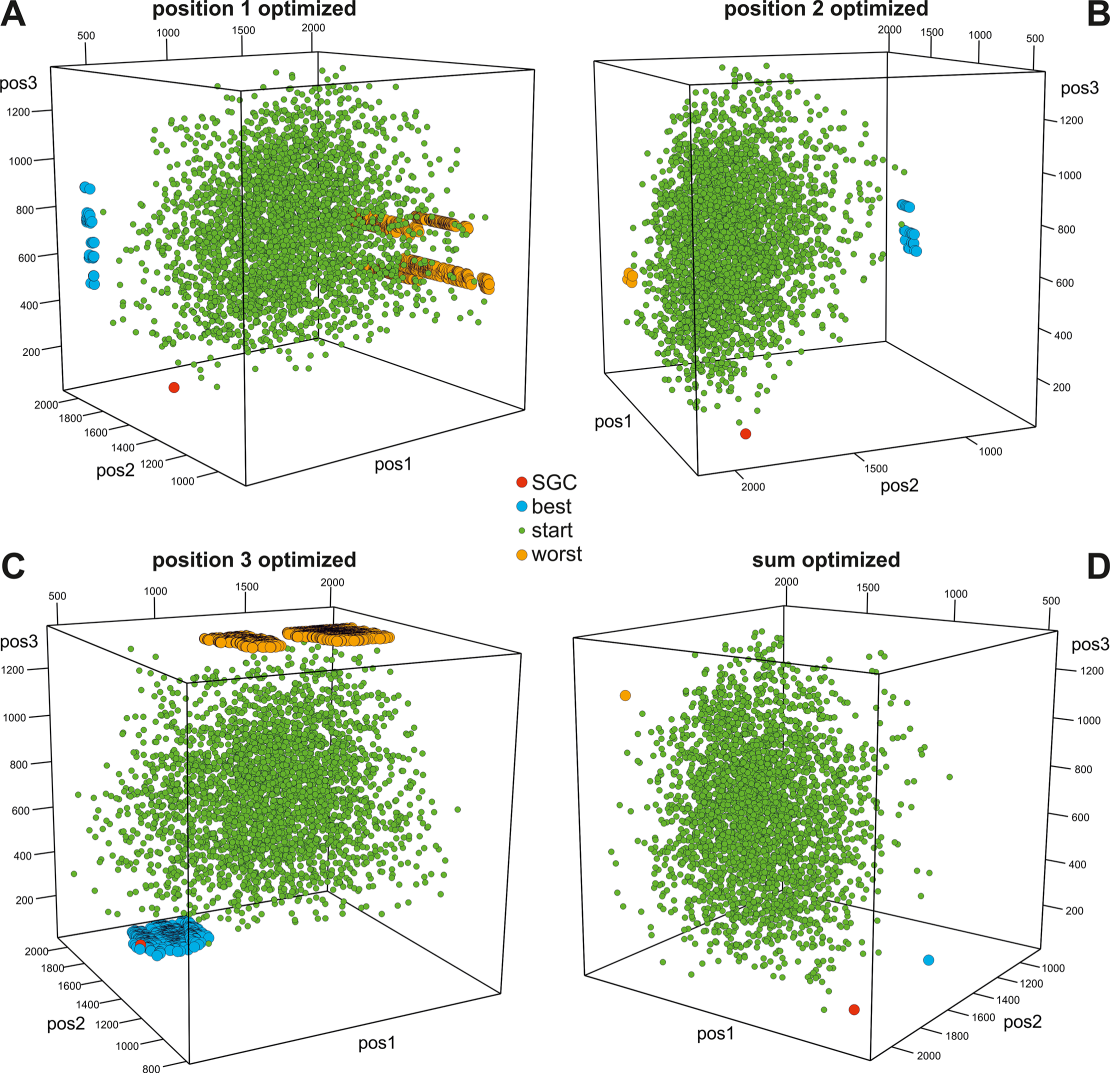
Graphical representation of the genetic codes in the three-dimensional space of objective function for three codon positions and the DEG model. The individual plots correspond to the scenarios in which polarity costs were optimized individually for one of the three codon positions (A, B, C) or for all of them (D). SGC, the standard genetic code; start, starting codes; best, the codes that minimize the objective function; worst, the codes that maximize the objective function. See S2–S5 Figs for interactive plots.

**Fig 4:**
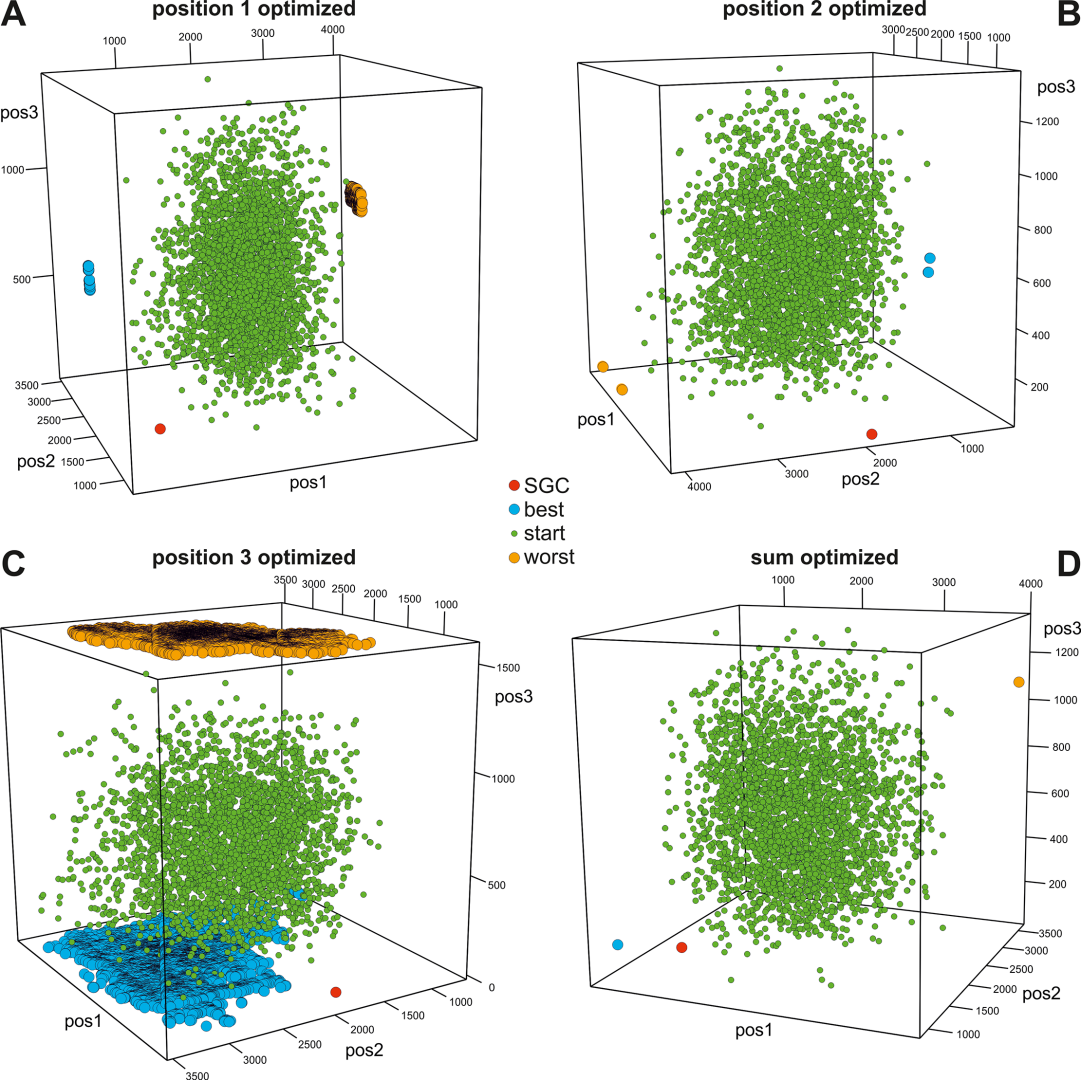
Graphical representation of the genetic codes in the three-dimensional space of objective function for three codon positions and the BLO model. See S6–S9 Figs for interactive plots. Other explanations are the same as those in Fig 3.

**Fig 5:**
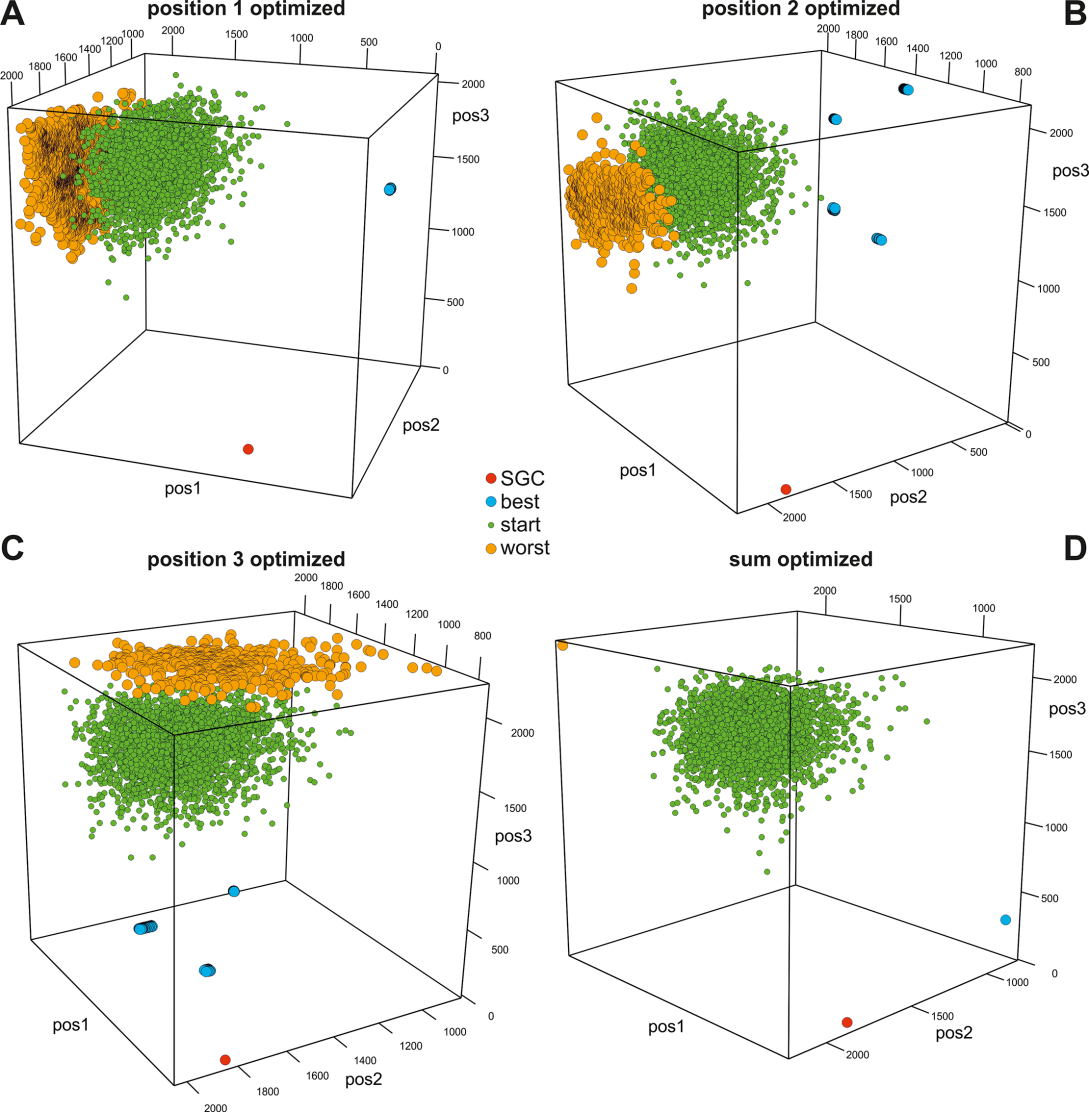
Distribution of genetic codes in the three-dimensional space of objective function for three codon positions and the NUM model. See S10–S13 Figs for interactive plots. Other explanations are the same as those in Fig 3.

**Fig 6:**
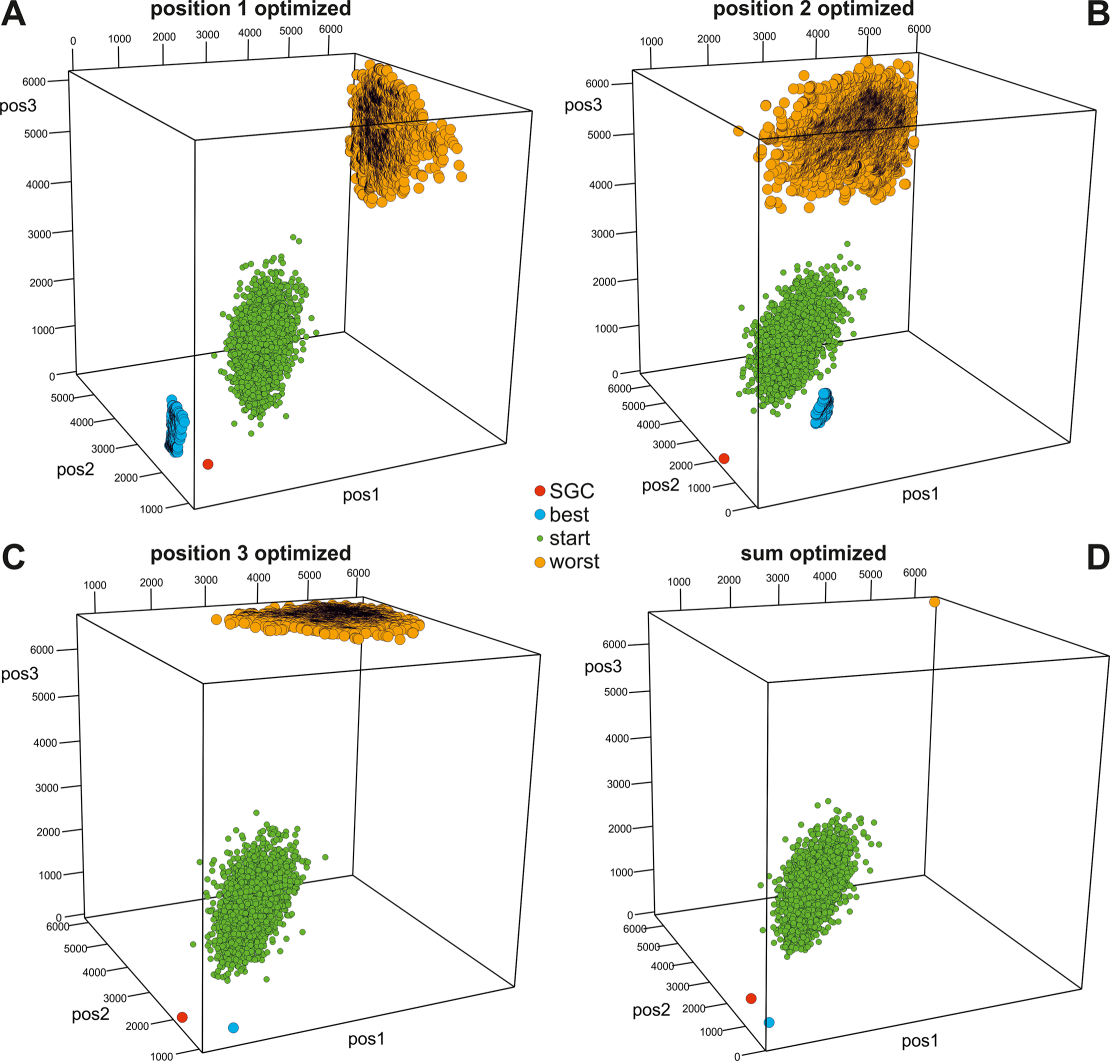
Graphical representation of the genetic codes in the three-dimensional space of objective function for three codon positions and the GEN model. See S14–S17 Figs for interactive plots. Other explanations are the same as those in Fig 3.

In Figs 3–6, we presented the distribution of the SGC, initial random codes and optimized codes in the three-dimensional space of the costs calculated for three codon positions individually in various optimization scenarios. Each of the figures (Figs 3–6) presents the results for one of the four genetic code models and includes four cases when the polarity costs were optimized individually for one of the three codon positions or for all positions. The corresponding interactive plots are included in S2–S17 Figs. We advise readers to use them for a better view of the positions of the genetic codes in the three-dimensional space of objective function.

The genetic codes that were optimized to minimize or maximize the polarity costs occupy extreme and opposite positions in the space (Figs 3–6). These codes can be represented by one solution, especially when the sum of the costs from all codon positions was optimized (Figs 3D–6D) or when many cases formed a layer of points, especially when the objective function of one codon position was optimized (e.g., Figs 4C, 5C and 6A). The randomly generated genetic codes that were used at the start of the genetic algorithm are located between these codes.

To describe the location of SGC in three-dimensional space relative to the best and worst genetic codes, we calculated the shortest Euclidean distances between the SGC and the best codes *ED*^best^ as well as between the SGC and the worst optimized codes *ED*^worst^, and we next subtracted these distances: *ED*^worst^−*ED*^best^. This measure differs from the *GD* in that it also takes into account the costs resulting from amino acid replacements in codon positions that were not directly optimized. In turn, *GD* considers only the optimized parameter.

**Fig 7:**
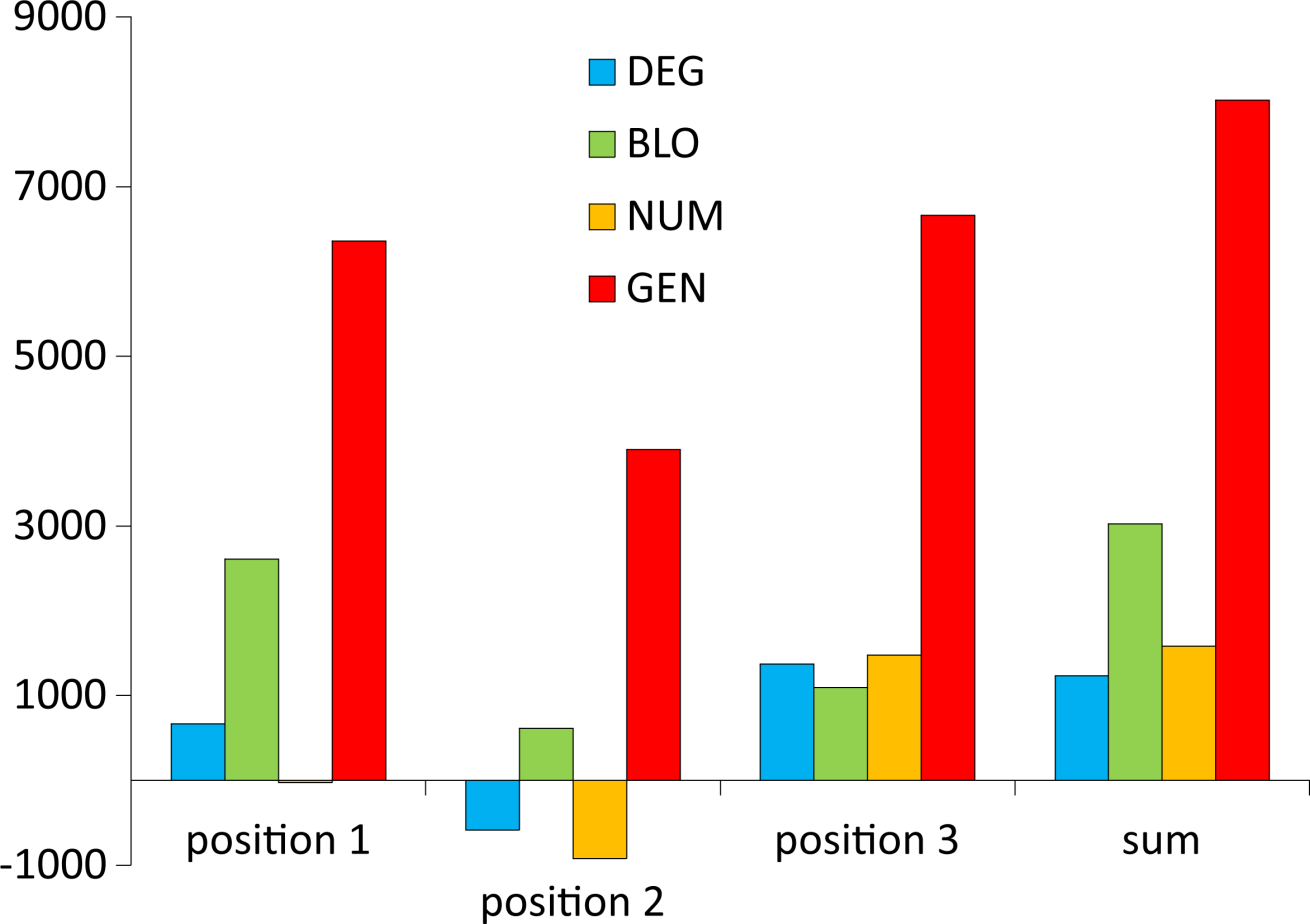
The difference *ED*^worst^−*ED*^best^, i.e., the closest Euclidean distances between the SGC and worst optimized codes (*ED*^worst^) and the SGC and best optimized codes (*ED*^best^), calculated under four models with different restrictions on the genetic code structure when the polarity costs were minimized for the three codon positions individually or as the sum of costs over all positions; GEN, the least restrictive model; NUM, the model preserving the number of codons per amino acid; BLO, the model preserving the codon block structure; DEG, the model preserving the degeneracy.

The position of the SGC in the space of the cost values depends on the optimized criterion and the assumed model of the genetic code (Fig 7). Generally, SGC is several times closer to the best code than to the worst code. The exceptions are the DEG and NUM models, where the costs in the second codon position were optimized (Fig 3B and Fig 5B, respectively). In these cases, the SGC is closer to the worst codes. In the case of the NUM model with the optimized first codon position, the SGC is almost equidistant to the extreme codes (Fig 5A). The zero distance between the SGC and the best code was only found for the DEG model when the cost for the third codon position was optimized. In this case, the SGC is located among the best codes (Fig 3C). The largest difference between the distances *ED*^best^ and *ED*^worst^ is for the general model (Fig 7). In this case, the SGC is quite close to the best codes at the global scale, especially when the sum of costs from all codon positions and the first and the third codon positions are optimized (Fig 6A, 6C and 6D). The *ED*^best^ is then more than five, eight and nine times larger than *ED*^worst^, respectively, for optimization of the costs from all codon positions and from the first and third codon positions. The SGC is also more than four and five times closer to the best than the worst code for the DEG and BLO models when the total cost was optimized (Figs 3D and 4D).

**Fig 8:**
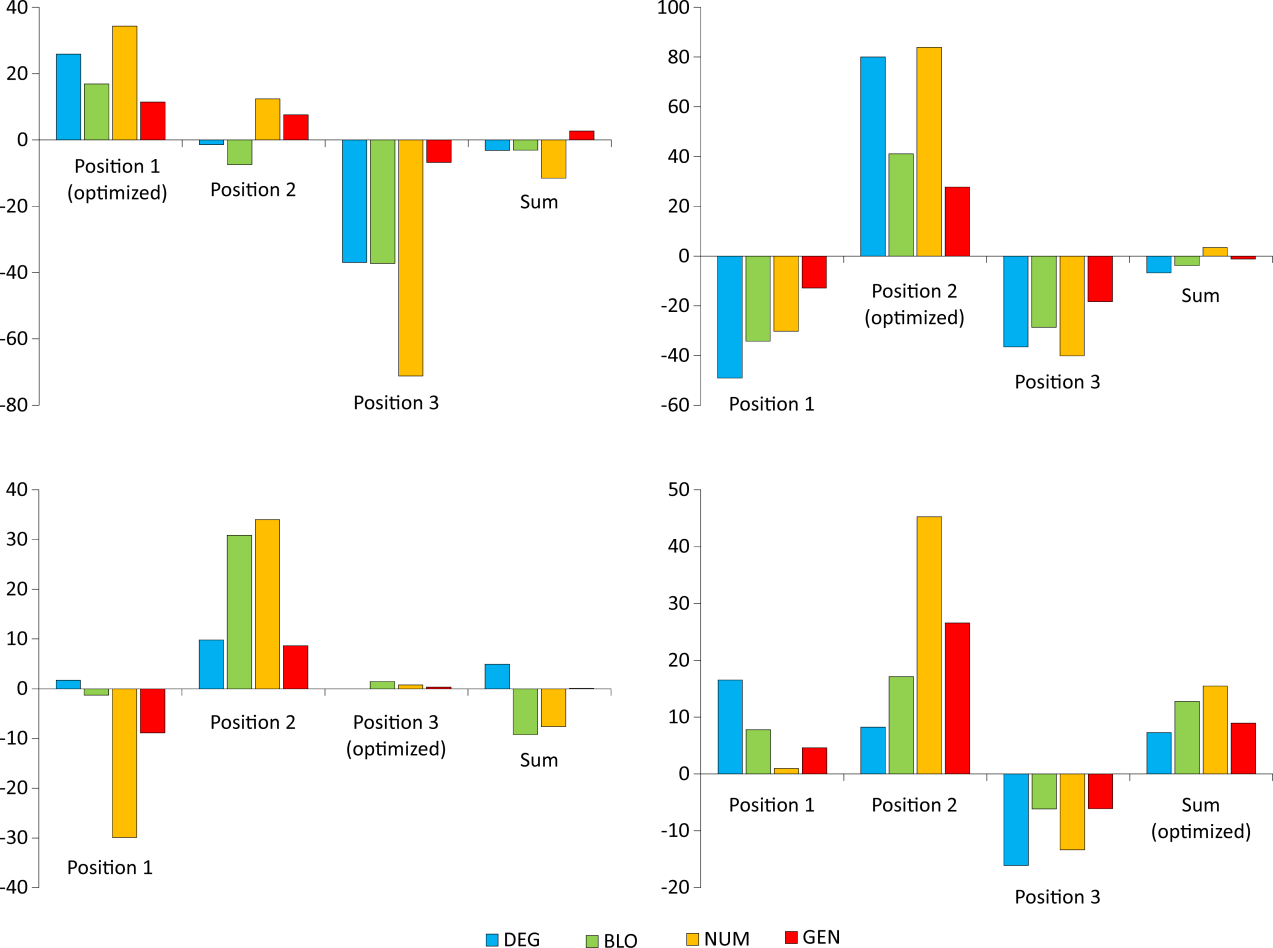
The *GD* measure calculated under four models of the genetic code (DEG, BLO, NUM, and GEN) when the polarity costs were minimized for three codon positions individually (A, B, and C) or as the sum of costs over all positions (D).

To easily compare and summarize these results for the non-optimized codon positions, we also calculated the general distance *GD* for the non-optimized codon positions and presented them together with the optimized ones in one chart for various genetic code models (Fig 8). In these calculations, the value for the standard genetic code *F*^SGC^ was subtracted from the minimum of objective function found for the non-optimized positions when the other position or the sum of costs over all positions was optimized. The denominator remained the same as for the *GD* distance of the optimized cases (Eq 3). Since we calculated the objective function for non-optimized positions, this parameter can be larger than the *F*^SGC^, and consequently, *GD* can be negative. A negative value indicates lower efficiency of the considered codon position in the minimization of amino acid replacement costs compared with the SGC. On the other hand, a positive value implies that a position that was not directly optimized has a better minimization ability than the position in the SGC.

Optimization of the genetic code to minimize the effects of amino acid replacements because of nucleotide substitutions in the first codon position is associated with the deterioration of minimizing properties in the third codon position for all models of the genetic code compared with the SGC. Accordingly, the *GD* for the third codon position ranges from −71.2% (for the NUM model) to −6.8% (for the GEN model) (Fig 8). However, optimization of the genetic code with respect to the first codon position has a smaller, model-dependent impact on the second codon position. Compared with the SGC, its objective function value is slightly larger (i.e., worse) in the best optimized codes for the most constrained models (DEG and BLO) and somewhat smaller (i.e., better) in the best codes for the NUM and GEN models. Then, the *GD* for the second codon position for these models is slightly negative (down to −7.4%) and positive (up to 12.4%), respectively, for the most constrained and least constrained models (Fig 8A). Similarly, the influence of the optimization according to the first codon position is also rather small on the sum of the costs in three codon positions. The *GD* of the sum decreases (down to −11.5%) for the DEG, BLO and NUM models and grows to 2.7% for the GEN model (Fig 8A).

Significant improvement of the genetic code in minimization of the amino acid replacement costs due to mutations in the second codon position is clearly associated with the worsening of such properties in the first and third codon positions. The *GD* values for the first second codon positions are negative, ranging from −12.8% to −49.1% (Fig 8B). However, the *GD* for the total cost in all codon positions does not change substantially compared with the SGC and ranges from −6.8% to 3.4% depending on the genetic code model (Fig 8B).

In contrast to the above-mentioned observations implying an inverse dependence between the second and third codon positions, optimization of the genetic code with respect to the third codon position improved in the second codon position. The *GD* values for the second codon position are positive, ranging from 8.7% to 34% (Fig 8C). However, optimization of the third codon position has a negative influence on optimization of the first codon position in the NUM and GEN models. In these cases, the *GD* values are negative, i.e., −29.9% and -8.9%, respectively, for the NUM and GEN models (Fig 8C). Still, the objective function values of the best codes optimized under the most restricted models (DEG and BLO) are comparable to the SGC with a *GD* 1.7% and −1.3%, respectively, for the DEG and BLO models (Fig 8C). Additionally, according to the *GD* measure calculated for the sum of costs over all codon positions, the SGC is better than the best codes under the BLO and NUM models with a *GD* of −9.3% and −7.6%, respectively (Fig 8C). Nevertheless, the SGC is slightly worse (*GD* = 4.9%) for the DEG model and comparable (*GD* = 0.1%) with the best codes in the GEN model (Fig 8C).

Optimization of the genetic codes regarding the total cost in all codon positions influences the individual positions regardless of the applied genetic code models (Fig 8D). Compared with the SGC, the first and second codon positions are improved in the best codes at the expense of the third position, which is less optimized than in the SGC with a *GD* ranging from −6.2% to −16.1%. The *GD* values range from 0.99% to 16.6% for the first codon position and from 8.3% to 45.3% for the second position (Fig 8D).

To conclude this part of our results, we can say that the SGC is still generally more similar to the codes minimizing the amino acid replacements than to those maximizing them when we also take into account the costs of codon positions that were not directly optimized. The similarity is greater under the general (unrestricted) model of the genetic code but smaller for restricted models. However, when the SGC is compared with the codes optimized for the costs in the second codon position under the DEG and NUM models, it is closer to the worst codes. The codes that were optimized to minimize amino acid replacements in an individual codon position in majority of cases are not optimal regarding all other individual positions or total costs. The minimization of cost for individual codon positions also has quite a small influence on the total costs over all positions in comparison to the SGC. However, the relationships are not straightforward, and they depend on the genetic code model. The most notable exceptions are the codes minimizing costs for the third position because they also minimize costs resulting from mutations in the second codon position better than the SGC. Among such codes optimized under the DEG model, it is also possible to find those codes that are slightly better than the SGC for the first codon position and the total costs. Moreover, the codes minimizing the sum of costs over all positions are also better than the SGC regarding the minimization of costs associated with the second and first codon positions. This finding implies that by minimizing costs for only one codon position, it is still possible to improve the SGC with respect to other positions. Therefore, the code is not fully optimized.

### Optimization properties of randomized genetic codes

**Fig 9:**
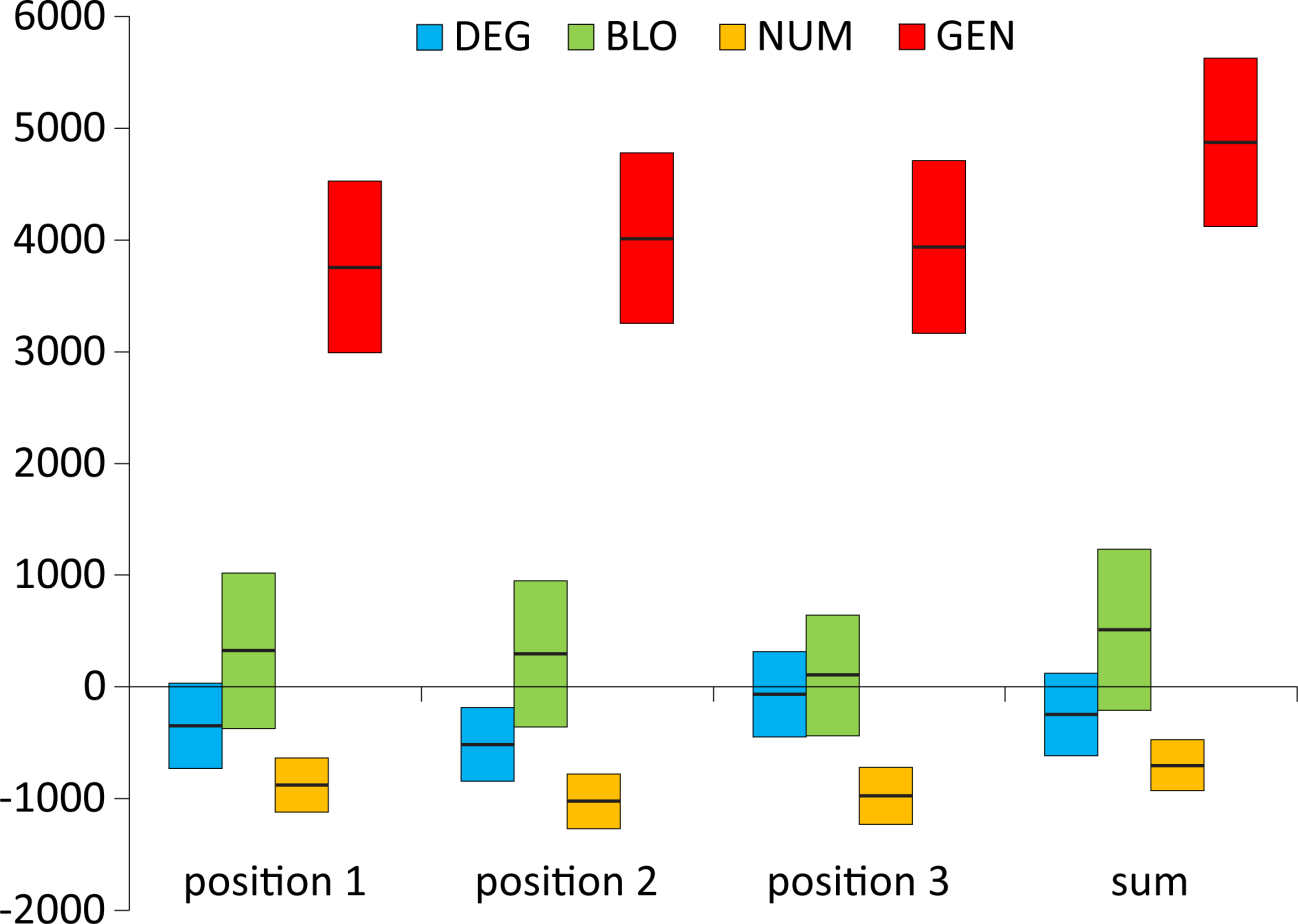
The difference $\bar{ED^{\mathrm{worst}}}-\bar{ED^{\mathrm{best}}}$, i.e., between the mean of the closest Euclidean distances of the randomized codes to the worst ($\bar{ED^{\mathrm{worst}}}$) and the best optimized codes ($\bar{ED^{\mathrm{best}}}$), calculated under four models with different restrictions on the genetic code structure when the polarity costs were minimized for the three codon positions individually or as the sum of costs over all positions; GEN, the least restrictive model; NUM, the model preserving the number of codons per amino acid; BLO, the model preserving the codon block structure; DEG, the model preserving the degeneracy. The range of box plots corresponds to two standard deviations and the thick horizontal line marks the mean.

One can assume that the randomly generated genetic codes should not show any optimization properties and ought to be placed roughly between the codes minimizing and maximizing the polarity costs in the centre of the three-dimensional space determined by the extreme values of the objective function for the optimized codes. Such locations can be observed for the DEG and BLO models of the genetic code, when the costs were optimized for both individual and all codon positions (Figs 3 and 4). Consequently, the difference between the average of the minimum Euclidean distances between the randomized codes and the worst ($\bar{ED^{\mathrm{worst}}}$) and best ($\bar{ED^{\mathrm{best}}}$) optimized ones is close to zero (Fig 9). The results do not depend on whether costs were optimized in individual or all codon positions. In contrast, the randomized codes are closer to the worst codes rather than best codes optimized under the NUM model (Fig 5). The range of two standard deviations does not encompass the zero value of the difference $\bar{ED^{\mathrm{worst}}}-\bar{ED^{\mathrm{best}}}$ for the NUM model (Fig 9). The difference is the greatest for the randomized codes obtained under the GEN model. In this case, the codes are much closer to the best codes than to the worst codes (Figs 6 and 9).

**Fig 10:**
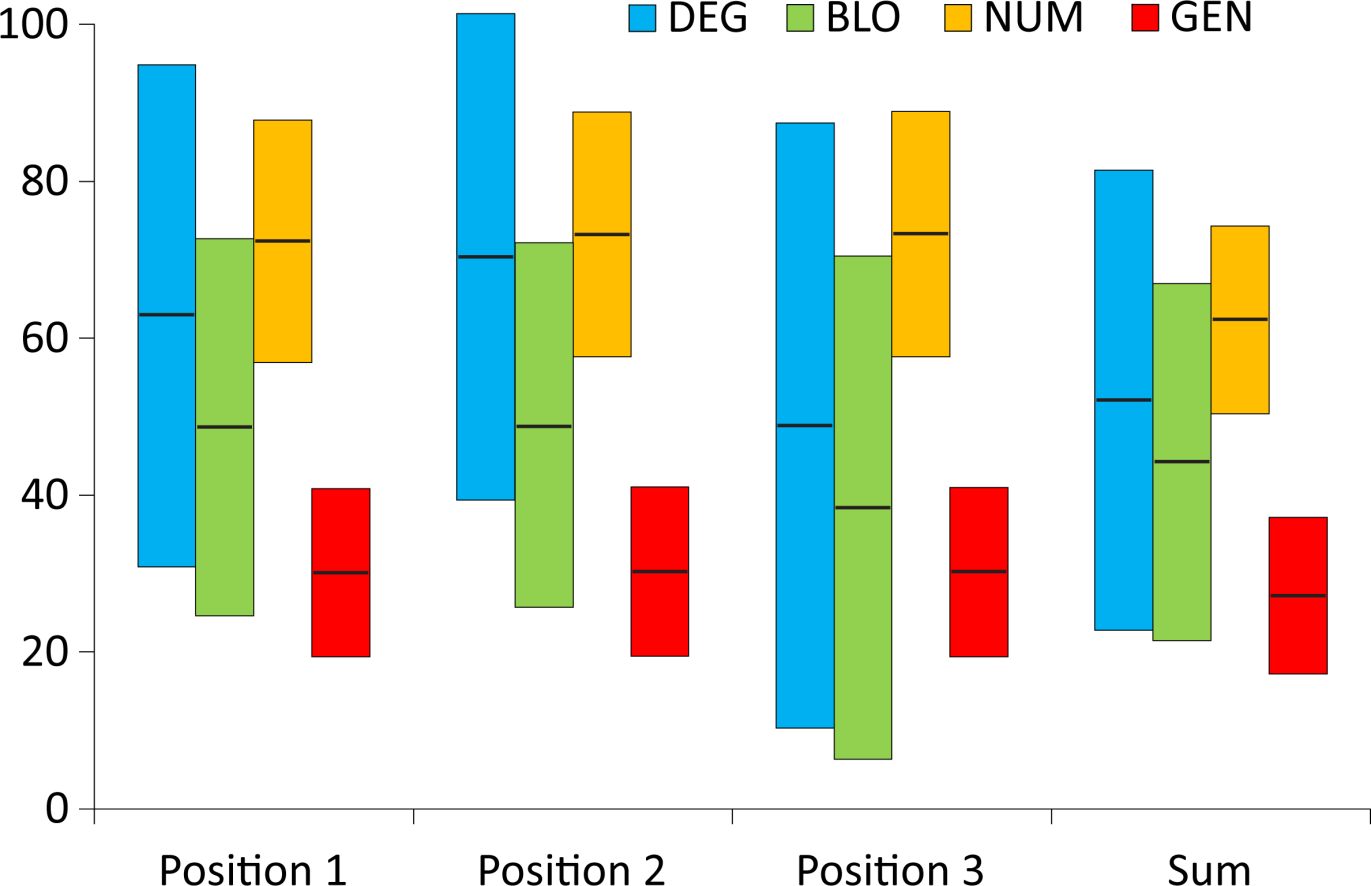
The global distance for the randomized codes, *GD*^rand^, calculated under four models with different restrictions on the genetic code structure when the polarity costs were minimized for the three codon positions individually or as the sum of costs over all positions; GEN, the least restrictive model; NUM, the model preserving the number of codons per amino acid; BLO, the model preserving the codon block structure; DEG, the model preserving the degeneracy. The range of box plots corresponds to two standard deviations and the thick horizontal line marks the mean.

Similar results were obtained for the global distance, *GD* (Fig 10). Assuming equidistant locations of the randomized codes to the codes minimizing and maximizing the polarity costs, we should expect their average *GD*^rand^ ≈ 50%. However, this measure shows a bias, which is the largest for the codes under the general model and is approximately 30%. It means that the randomized codes for this model show a greater tendency to minimize rather than to maximize the polarity costs.

This result implies also that the random codes generated by the assignment of 20 amino acids with equal probabilities to at least one of the 61 codons may show a tendency to minimize the amino acid replacement costs when compared with the extremely bad codes. This observation has some interesting implications for the evolution of the standard genetic code. If the initial code were created in a similar way to the random codes, it had at the initial stage of its evolution, a greater inclination to minimize than to maximize the costs of amino acid replacements. However, a different conclusion about the optimality of the SGC may be drawn for another group of random codes depending on the randomization method and the selected reference set.

### Comparison of the genetic code structures

**Fig 11:**
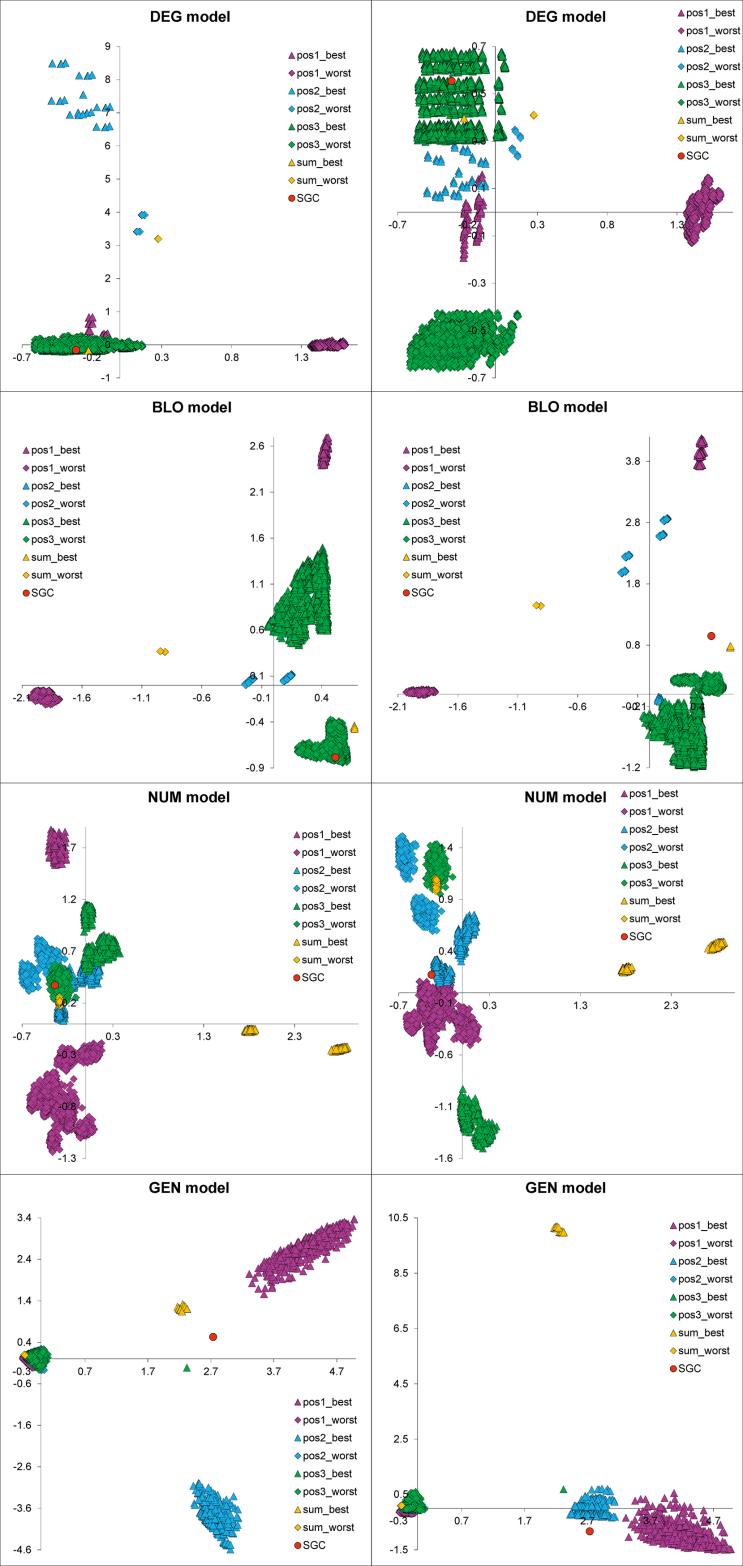
The plots of correspondence analysis comparing the structures of the genetic codes for the first and second components (the left-hand panel) and the first and third components (the right-hand panel). The prefixes pos_1, pos_2, pos_3 and sum indicate that the given code was optimized to minimize (best) or maximize (worst) the objective function according to the amino acid replacement costs in the first, second, and third codon positions as well as the total costs for the three codon positions, respectively. The plots are shown separately for four models with different restrictions on the genetic code structure: GEN, the least restrictive model; NUM, the model preserving the number of codons per amino acid; BLO, the model preserving the codon block structure; DEG, the model preserving the degeneracy. See S18–S21 Figs for interactive plots.

Besides the quantitative assessment of SGC optimality in comparison to the optimized genetic codes, it is also interesting to check how many assignments of amino acids to codons are shared by the studied codes. To that end, we expressed the codes as binary strings in which 1 and 0 indicated the presence or absence of a given assignment, respectively. Such representations were visualized in the plots of correspondence analysis (CA) (Fig 11, see also interactive plots in S18–S21 Figs). The optimization algorithm found many codes that were optimized under a given criterion and had the same value of the objective function but differed in the structure. Almost all sets of optimized codes under the DEG and BLO models form separate and coherent groups, which implies their different and specific structures. In the case of the NUM model, some codes optimized in the same way consist of separate subsets. This finding suggests that the same level of optimality can be achieved by codes with somewhat different structures. The separate groups are clearly visible in the interactive plot in S18–S21 Figs. On the other hand, the codes that maximized the costs for any codon position and the total costs under the GEN model were tightly packed together in contrast to the codes minimizing polarity costs. This result indicates that the worst codes share relatively similar structures under the relaxed model of the genetic code.

In the CA plots that included codes optimized under the DEG model, the SGC is located among the codes minimizing costs in the third codon position and is close to the code minimizing the sum of costs in all three codon positions (Fig 11, see also interactive plots in S18–S21 Figs). The codes of the latter type are also the closest to the SGC in the BLO model. Interestingly, the next group closest to the SGC includes the codes that maximized costs in the third codon position under the BLO model. The SGC is near the codes minimizing costs in the first codon position under the NUM model, whereas for the GEN model, the SGC is placed between the codes minimizing costs in the first and third codon positions. The presented CA plots can be used only for general comparison of the code structures because even the three visualized dimensions do not explain a large fraction of the variance in the sets.

Therefore, to fully compare the code structures, we introduced the structure distance *SD*, which ranges from 0% to 100% and can be understood as the percentage of different assignments of amino acids to codons in the compared codes. Using this measure, it is possible to identify the optimized codes to which the SGC is most similar regarding its structure. These results are comparable to the CA plots. However, the value of the structural difference between the SGC and the optimized codes is generally large for the BLO, NUM and GEN models, and there is no great difference in the *SD* for various optimized scenarios (Table 1). In most cases, the minimum *SD* is larger than 80% and on average greater than 90%. Similar average values were received in the comparison of the SGC with randomized codes: 93.7% for the NUM model and 95% for the BLO and GEN models.

**Table 1:** The structure distance *SD* between the standard genetic code and the optimized (best and worst) codes.

Model	Optimized parameter	Best code			Worst code		
		Mean	Min	Max	Mean	Min	Max
DEG	pos1	36.0	23.0	49.2	80.6	65.6	88.5
	pos2	90.2	85.2	95.1	68.9	62.3	75.4
	pos3	53.3	3.3	75.4	66.2	26.2	85.2
	sum	47.5	45.9	49.2	77.0	72.1	82.0
BLO	pos1	99.2	93.4	100	98.4	93.4	100
	pos2	100	100	100	90.2	80.3	100
	pos3	98.4	83.6	100	89.1	44.3	100
	sum	83.6	77.0	90.2	100	100	100
NUM	pos1	88.7	82.0	93.4	94.7	86.9	100
	pos2	92.3	86.9	98.4	92.7	86.9	98.4
	pos3	94.7	88.5	100	96.3	90.2	100
	sum	98.9	95.1	100	93.4	91.8	95.1
GEN	pos1	91.5	82.0	98.4	94.4	85.2	100
	pos2	93.7	86.9	100	95.7	86.9	100
	pos3	91.8	91.8	91.8	95.5	88.5	100
	sum	100	100	100	95.9	93.4	98.4

Much smaller values of *SD* were observed for the DEG model, which may be associated with its much stricter rules on code structure (Table 1). This model preserves both the degeneracy and codon block structure. The *SD* values show also greater variation and depend more on the optimized criteria in the DEG model than under the other models. The SGC structure is more similar to the codes minimizing polarity costs than maximizing polarity costs in the first and third codon positions as well as those minimizing the total costs over all positions. The mean *SD* for such comparisons is 36%, 53% and 48%, respectively, for the first codon position, third codon position, and all codon positions. Among the codes minimizing the costs in the first codon position, it is possible to find the code that differs from the SGC in only 23% of assignments. The code most similar to the SGC minimizes the costs in the third codon position and differs from the SGC only in 3%. Interestingly, among this group of optimized codes sharing the same value of the objective function, a code whose structure differs from the SGC by more than 75% exists. The codes maximizing the costs in the second codon positions are more similar to the SGC in structure than the codes minimizing these costs: the minimum *SD* = 62% and 85%, respectively. The codes randomized under the DEG model show on average 73.7% structural difference to the SGC.

The presence of optimized codes with the same value of the objective function but with different structures implies that the same level of optimality can be realized by various assignments of amino acids to codons. In agreement, Spearman correlation coefficients between the structure distances *SD* and the Euclidean distances in the objective function values *ED* between the SGC and the optimized codes are low. This coefficient is in the range of -0.049 to 0.097 for the BLO, NUM and GEN models optimized for costs in individual and all codon positions. Some positive correlations that were slightly higher were observed for the DEG model (0.155–0.282), but the value is still quite low.

In S1 Table, we compared directly the tables of the SGC and the optimized genetic codes that are most similar to the SGC according to structure, i.e., showing the minimum *SD*. The assignments of amino acids to codons typical of the SGC and present in the optimized codes were also highlighted. When several different codes showed the same minimum *SD*, the most frequent amino acid assigned to a codon was shown together with the percentage of its occurrence in the codes. The distribution of the assignments depends on the restrictions imposed on the genetic code model and the codon position that was selected to optimize amino acid replacement costs as a result of nucleotide substitutions in this position.

The codes that are not restricted to the block structure of the SGC, i.e., the NUM and GEN models, and minimize the objective function for the substitutions in the first codon position have the same or similar amino acids assigned for codons that differ only in the first codon position (S1 Table). Accordingly, every fourth codon in the columns of the genetic code table has the same or a similar amino acid. When the consequences of substitutions in the second codon position are minimized, amino acids with a similar polarity are arranged in rows of the genetic code table, and if the objective function for the third codon position is minimized, a characteristic codon block structure appears in the table. For example, under the most general model (GEN), there are two amino acids (Pro and Tre) coded by two codons, four amino acids (Asn, His, Leu, and Met) coded by three codons, seven amino acids (Arg, Cys, Gly, Ser, Trp, Tyr, and Val) coded by four codons and one (Ala) amino acid coded by eight codons. The codes that optimize the total cost of amino acid replacements resulting from nucleotide substitutions in all three codon positions show a ‘mixed’ or ‘compromised’ arrangement of amino acids in the genetic code table of the NUM and GEN models. Therefore, it is possible to recognize amino acids with similar properties in rows, columns and codon blocks. The best code minimizing the total cost under the GEN model is characterized by as many as 18 codons for Ala: 16 MNS codons and two TYA codons, where M = A or C, S = G or C and N = A, T, G or C (S1 Table). It means that in this case the second codon position is the most degenerated one, however both the first and the third positions are also degenerate but for different types of nucleotides. A similar degeneration scheme is presented by Ser codons: 8 GNS codons and 4 MYT codons. In turn, Gly codons are: 6 KYB and 4 MRT, where: K = G or T, B = G, C or T, R = A or G. In contrast, the codes that maximized the amino acid replacements did not show such specific arrangements of amino acids with similar polar properties.

Only a few assignments of amino acids to codons in the SGC match those in the best genetic codes found under the NUM and GEN models (S1 Table). Most of the common assignments are scattered over the whole genetic code table, and usually, they do not form the typical codon block structure present in the SGC. Several codon blocks typical of the SGC can be recognized in the optimized codes. For example, two blocks consisting of two codons for Arg can be found in the code that minimizes the objective function for the first codon position. A two-codon block for Ser and a three-codon block for Leu appear in the code minimizing the cost for the third codon position. Two blocks were also found in the code that maximizes the costs in the second codon position: a three-codon block for Ile and a two-codon block for Asp.

The two other genetic code models, DEG and BLO, assume the block structure of the SGC. Therefore, the presence of some codon blocks matching those in the SGC are expected. However, in the case of the less restricted BLO model, the number of such matches does not exceed half of all 22 codon blocks present in the SGC and is usually not greater than four (S1 Table). The best codes under the most restricted DEG model have the largest number of the amino acid assignments present in the SGC. The code minimizing the sum of cost for all three codon positions has the same codon blocks as the SGC for Phe, Leu, Ile, Met, Val, Ser, Tyr, Cys, Trp and Arg. The DEG codes that minimize the objective function for the first and third codon positions have more codon blocks matching those in the SGC than the maximizing codes (15 vs 9 and 20 vs 11, respectively). The code minimizing the polarity costs for the substitutions in the third codon position differs from the SGC only in the assignment of two one-codon amino acids: Met to TGG and Trp to ATG. On the other hand, the code of the BLO model having the largest number of blocks matching those in the SGC, i.e., 10, maximizes the cost for the third codon position. Its counterpart minimizing the costs for the third codon position has only three such matches. Similarly, the worst codes optimized for the second codon position have more amino acid assignments matching those in the SGC than the best codes both for the DEG (9 vs 4) and BLO model (3 vs 0). Nevertheless, when the total cost for all codon positions is considered, the best codes contain more matching blocks than the worst ones, 12 vs 7 and 4 vs 0, for the DEG and BLO models, respectively.

Concluding this part of the results, we can state that the same minimum level of optimization regarding the polarity costs can be achieved by various codes with different assignments of amino acids to codons. It implies quite a large flexibility of genetic code structure. However, the standard genetic code shows very poor similarity to the structure of the optimized codes. Therefore, to induce a substantial increase in the optimality of the SGC regarding the individual codon positions or all of them together, it is necessary to change over 80% of the assignments in most cases. Only keeping the degeneracy and codon blocks in the optimized codes the same as in the SGC makes the SGC structure similar to the codes minimizing the polarity costs in the third and first codon positions. In other words, the structure of the SGC is quite well optimized in only its own class of codes but not in general.

## Discussion

The sum of polarity costs for all three codon positions is optimized to a certain degree in the SGC, which suggests that some robustness was favoured against the effects of the point mutations in RNA or DNA coding for peptides or proteins. However, the differentiated level of the redundancy and optimization of the three codon positions have to result from specific mechanisms or processes that recognize these positions. The second codon position is the least redundant and the third codon position is the most redundant. This qualitative feature was assessed by us in a quantitative approach, in which we estimated the optimization of the individual codon positions in terms of amino acid replacements regarding their polar properties. The analyses clearly showed that the individual codon positions are variously optimized in terms of the cost of amino acid replacements measured by differences in their polar properties. The third codon position of the SGC is the best in minimizing these costs under all considered models of the genetic code, whereas the second position is the least optimized. In the case of the most general model of the genetic code (GEN), the first codon position is two and a half times better optimized in this respect than the second codon position, whereas the third codon position is 81 times better optimized than the second codon position. Simultaneously, the third position minimizes the amino acid replacement costs 34 times more effectively than the first one.

If the SGC had evolved to minimize the effects of mutations occurring in DNA or RNA due to replication or transcription processes, we would instead expect a similar robustness of the codon positions against mutations because such changes occur randomly regardless of the positions in the nucleic acids. Therefore, other mechanisms differentiating these positions must have participated in the evolution in the SGC structure.

One of the mechanisms can be associated with different translational fidelity of the individual codon positions. Therefore, we should expect that a position more susceptible to mismatches or allowing nonconventional base pairing should be better optimized to minimize the consequences of mistranslations at the amino acid level. According to this view, the second codon position should be less error-prone than the third one as indicated by optimization analyses. In fact, the possibility of nonconventional pairing between bases in the third codon position and the first anticodon position is commonly known as wobble paring [52], which is extended due to chemical modifications of bases [66, 67]. The relatively high robustness to costs of amino acid replacements in the first codon position compared with the second position could suggest that the first position was also ambiguously read in the past as the third one today. However, experimental analyses do not provide consistent conclusions about the translational fidelity of all three codon positions in general, and the results depend on the experimental model applied [see the comments by David Ardell on the paper by Novozhilov, Wolf [21] and references therein]. Moreover, wobble paring in the third codon position could have evolved to limit the amount of tRNAs recognizing synonymous codons. It would decrease the number of genes coding for tRNAs and, consequently, decrease the genome size, which would shorten the time and reduce the costs of genome replication [68–70]. Such genomes may have a greater selective advantage. Thereby, the high optimization level of the third codon position could be a by-product of this process.

The other explanation for the diverse optimization level of the codon positions is provided by the 2-1-3 model [25, 71] and the four-column theory [22], which assume that the second base of the codon was the first to code information about amino acids, whereas the other positions were irrelevant. Next, the specificity of the coding was extended to the first base and then to the third base in the codon. Since the singlet codons with only the second base informative differed from each other in a single point mutation or mistranslation, it was not possible to minimize errors in this position [71]. Only the inclusion of the first codon position in a doublet code gave a possibility to assign amino acids with similar physicochemical properties to codons that differed in only one position. A triplet code involving the third position for encoding offered more possibilities of such assignments and then the greater flexibility of the third codon position in error minimization. The potential optimization of the second codon position might have been possible by changing already established assignments of amino acids to codons, but it would have required recoding many proteins and peptides already accepted by selection. The different optimization level of the codon positions would be a by-product of: (i) the increasing coding specificity of the individual positions, as assumed in the 2-1-3 model [25, 71], or (ii) the special additions of amino acids to the code to minimize the disruption of already existing genes, as the four-column theory postulates [22].

The greater conservatism of the second codon position may also be associated with constraints on interactions between base pairs in RNAs and their structure stability [22, 72, 73]. If only two pairs of bases interact between a codon and an anticodon, the structure is much more stable if the paired bases are adjacent, i.e., the middle codon position interacts with the middle anticodon position and the first codon position interacts with the third anticodon position or the third codon position interacts with the first anticodon position. When the paired bases are separated, i.e., only the first codon position interacts with the third anticodon position and the third codon position interacts with the first anticodon position, the structure is less stable. Consequently, a mismatch or mutation in the middle base would result in an unstable interaction, and an error in a translation would not occur. In contrast, a mismatch involving the first or the third codon position would still leave two neighbouring base pairs, and a translation of such codon would occur. Therefore, one can expect an evolution of the code that minimizes the consequences of changes in the first and the third codon positions. In fact, the optimization of these positions is much larger in the first and third positions than in the second one.

The adaptive hypothesis seems very attractive because it considers the SGC as a self-optimization system that evolved to minimize the effects of amino acid replacements [5–9, 11, 13, 51, 74–76]. We also found that the SGC is quite well optimized at the global scale, including both the best and worst codes, even up to 9% for the unrestricted model, but only when the sum of costs for all codon positions is considered. Its robustness is much weaker to the replacements in the first and the second positions individually. It results from the fact that the codes compared with the SGC and optimized for all codon positions simultaneously cannot have all the individual position perfectly optimized. The optimization of one positions involves the deterioration of another. If we want to obtain a code that is optimized in general, i.e., minimizes the sum of costs in all three codon positions, we should significantly improve such properties in the first and second codon positions at the expense of the third one compared with the SGC. We also found that the codes minimizing the costs in only one codon position, i.e., the third or first one, outperform the SGC in the second codon position. Thus, there is something to improve in the SGC. Our results are in agreement with some other studies showing that this code is only partially optimized or even is not located at a local minimum [21, 23, 62]. Therefore, it is possible to find much better codes.

Our results also demonstrate that the structure of the genetic code differs considerably from the most optimized codes. We also found that it is possible to generate many equivalent codes with the same level of optimization but with different assignments of amino acids to codons. This finding implies that the structures of the genetic codes have many degrees of freedom. What is more, by comparing randomly generated codes with the best and worst ones, we discovered that most of the random codes show a tendency to minimize the costs of amino acid replacements under some of the genetic code models. In other words, if the SGC had evolved by roughly uniform assignment of amino acids per codons, it would have had a large chance to obtain this property at the beginning.

To conclude all of our findings, we can state that the SGC was not directly optimized to minimize the consequences of mutations and mistranslations and its optimization properties can be a by-product of other mechanisms. One of them could have been the code expansion associated with the duplication of genes for adaptor molecules (proto-tRNAs) and charging enzymes (proto-aaRS) [25–27, 47, 77]. These mechanisms reconcile the properties of the SGC with the ‘frozen accident’ concept assuming that amino acids were assigned to codons initially by chance and that after fixation of these assignments, no substantial changes were allowed because they would be lethal [2]. Alternatively, the amino acids could have been added to the code in accordance with biosynthetic pathways and the physico-chemical properties of amino acids could have played only a subsidiary role in the structuring of the genetic code [34, 35]. Moreover, the damaging effects of mutations could have been minimized by other processes directly influencing mutational pressure without requiring any reorganization of the genetic code [12, 78, 79].

## Supporting information

S1 Fig Effects of amino acid replacements as function of generations for 20 independent runs for the general model of the genetic code. The objective function was minimized for the three codon positions and the sum of costs for the all codon positions.
(PDF)

S2 Fig Interactive plot showing genetic codes in the three-dimensional space of the objective function for three codon positions under the DEG model and optimized costs in the first codon position. SGC, the standard genetic code; start, starting codes; best, the codes that minimize the objective function; worst, the codes that maximize the objective function.
(HTML)

S3 Fig Interactive plot showing genetic codes in the three-dimensional space of the objective function for three codon positions under the DEG model and optimized costs in the second codon position. SGC, the standard genetic code; start, starting codes; best, the codes that minimize the objective function; worst, the codes that maximize the objective function.
(HTML)

S4 Fig Interactive plot showing genetic codes in the three-dimensional space of the objective function for three codon positions under the DEG model and optimized costs in the third codon position. SGC, the standard genetic code; start, starting codes; best, the codes that minimize the objective function; worst, the codes that maximize the objective function.
(HTML)

S5 Fig Interactive plot showing genetic codes in the three-dimensional space of the objective function for three codon positions under the DEG model and optimized the sum of costs in all codon positions. SGC, the standard genetic code; start, starting codes; best, the codes that minimize the objective function; worst, the codes that maximize the objective function.
(HTML)

S6 Fig Interactive plot showing genetic codes in the three-dimensional space of the objective function for three codon positions under the BLO model and optimized costs in the first codon position. SGC, the standard genetic code; start, starting codes; best, the codes that minimize the objective function; worst, the codes that maximize the objective function.
(HTML)

S7 Fig Interactive plot showing genetic codes in the three-dimensional space of the objective function for three codon positions under the BLO model and optimized costs in the second codon position. SGC, the standard genetic code; start, starting codes; best, the codes that minimize the objective function; worst, the codes that maximize the objective function.
(HTML)

S8 Fig Interactive plot showing genetic codes in the three-dimensional space of the objective function for three codon positions under the BLO model and optimized costs in the third codon position. SGC, the standard genetic code; start, starting codes; best, the codes that minimize the objective function; worst, the codes that maximize the objective function.
(HTML)

S9 Fig Interactive plot showing genetic codes in the three-dimensional space of the objective function for three codon positions under the BLO model and optimized the sum of costs in all codon positions. SGC, the standard genetic code; start, starting codes; best, the codes that minimize the objective function; worst, the codes that maximize the objective function.
(HTML)

S10 Fig Interactive plot showing genetic codes in the three-dimensional space of the objective function for three codon positions under the NUM model and optimized costs in the first codon position. SGC, the standard genetic code; start, starting codes; best, the codes that minimize the objective function; worst, the codes that maximize the objective function.
(HTML)

S11 Fig Interactive plot showing genetic codes in the three-dimensional space of the objective function for three codon positions under the NUM model and optimized costs in the second codon position. SGC, the standard genetic code; start, starting codes; best, the codes that minimize the objective function; worst, the codes that maximize the objective function.
(HTML)

S12 Fig Interactive plot showing genetic codes in the three-dimensional space of the objective function for three codon positions under the NUM model and optimized costs in the third codon position. SGC, the standard genetic code; start, starting codes; best, the codes that minimize the objective function; worst, the codes that maximize the objective function.
(HTML)

S13 Fig Interactive plot showing genetic codes in the three-dimensional space of the objective function for three codon positions under the NUM model and optimized the sum of costs in all codon positions. SGC, the standard genetic code; start, starting codes; best, the codes that minimize the objective function; worst, the codes that maximize the objective function.
(HTML)

S14 Fig Interactive plot showing genetic codes in the three-dimensional space of the objective function for three codon positions under the GEN model and optimized costs in the first codon position. SGC, the standard genetic code; start, starting codes; best, the codes that minimize the objective function; worst, the codes that maximize the objective function.
(HTML)

S15 Fig Interactive plot showing genetic codes in the three-dimensional space of the objective function for three codon positions under the GEN model and optimized costs in the second codon position. SGC, the standard genetic code; start, starting codes; best, the codes that minimize the objective function; worst, the codes that maximize the objective function.
(HTML)

S16 Fig Interactive plot showing genetic codes in the three-dimensional space of the objective function for three codon positions under the GEN model and optimized costs in the third codon position. SGC, the standard genetic code; start, starting codes; best, the codes that minimize the objective function; worst, the codes that maximize the objective function.
(HTML)

S17 Fig Interactive plot showing genetic codes in the three-dimensional space of the objective function for three codon positions under the GEN model and optimized the sum of costs in all codon positions. SGC, the standard genetic code; start, starting codes; best, the codes that minimize the objective function; worst, the codes that maximize the objective function.
(HTML)

S18 Fig Interactive plot of correspondence analysis for the first three components comparing the structures of the genetic codes under the DEG model. The prefixes pos_1, pos_2, pos_3 and sum indicate that the given code was optimized to minimize (best) or maximize (worst) the objective function according to the amino acid replacement costs in the first, second, and third codon positions as well as the total costs for the three codon positions.
(HTML)

S19 Fig Interactive plot of correspondence analysis for the first three components comparing the structures of the genetic codes under the BLO model. The prefixes pos_1, pos_2, pos_3 and sum indicate that the given code was optimized to minimize (best) or maximize (worst) the objective function according to the amino acid replacement costs in the first, second, and third codon positions as well as the total costs for the three codon positions.
(HTML)

S20 Fig Interactive plot of correspondence analysis for the first three components comparing the structures of the genetic codes under the NUM model. The prefixes pos_1, pos_2, pos_3 and sum indicate that the given code was optimized to minimize (best) or maximize (worst) the objective function according to the amino acid replacement costs in the first, second, and third codon positions as well as the total costs for the three codon positions.
(HTML)

S21 Fig Interactive plot of correspondence analysis for the first three components comparing the structures of the genetic codes under the GEN model. The prefixes pos_1, pos_2, pos_3 and sum indicate that the given code was optimized to minimize (best) or maximize (worst) the objective function according to the amino acid replacement costs in the first, second, and third codon positions as well as the total costs for the three codon positions.
(HTML)

S1 Table The tables of the SGC and the optimized genetic codes that are most similar to the SGC according to the structure, i.e. showing the minimum *SD*. The assignments of amino acids to codons typical of the SGC and present in the optimized codes were highlighted in yellow. When several different codes showed the same minimum *SD*, the most frequent amino acid assigned to a codon was shown together with the percentage of its occurrence in the codes. In this case, the assignments of amino acids to codons being the same as in the SGC were highlighted in light yellow.
(XLSX)
