# Correction: Optimization of the standard genetic code according to three codon positions using an evolutionary algorithm

**DOI:** 10.1371/journal.pone.0205450

**Published:** 2018-10-04

**Authors:** Paweł Błażej, Małgorzata Wnętrzak, Dorota Mackiewicz, Paweł Mackiewicz

There is an error in the second sentence of the fourteenth paragraph of the Results section. The correct sentence is: The GD values for the first and third codon positions are negative, ranging from −12.8% to −49.1% (Fig 8B).

In Fig 8, the headings are missing. Please see the corrected Fig 8 here.

**Fig 8:**
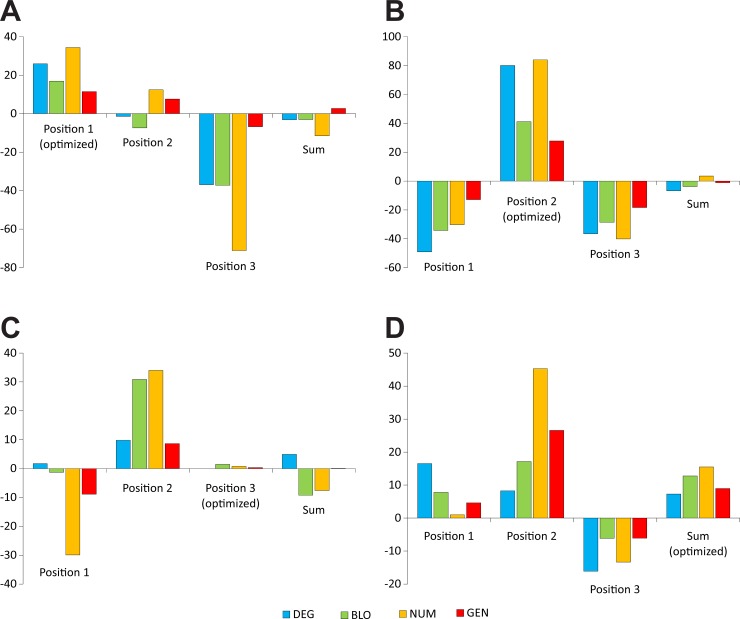
The *GD* measure calculated under four models of the genetic code (DEG, BLO, NUM, and GEN) when the polarity costs were minimized for three codon positions individually (A, B, and C) or as the sum of costs over all positions (D).
