# Supplementary figures and images for: Optimization of the standard genetic code according to three codon positions using an evolutionary algorithm
Source: PLoS One. 2018 Aug 9;13(8):e0201715. doi: 10.1371/journal.pone.0201715 (PMC6084934; doi:10.1371/journal.pone.0201715)

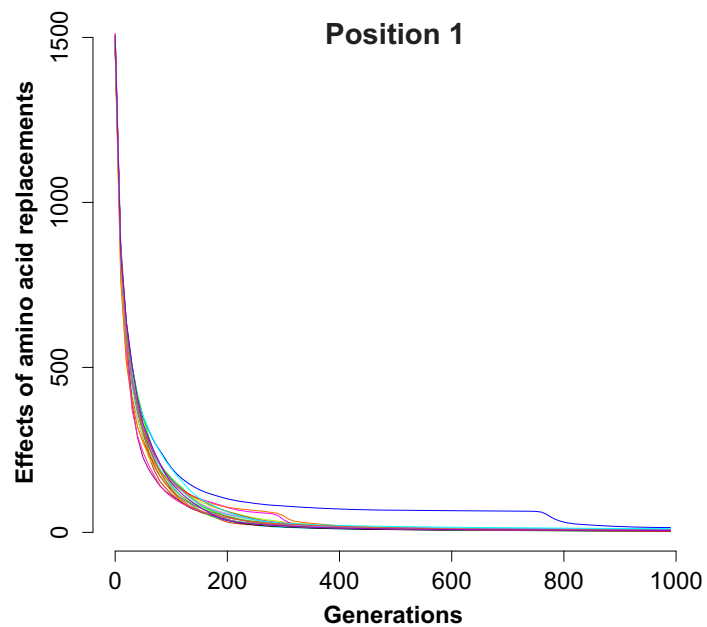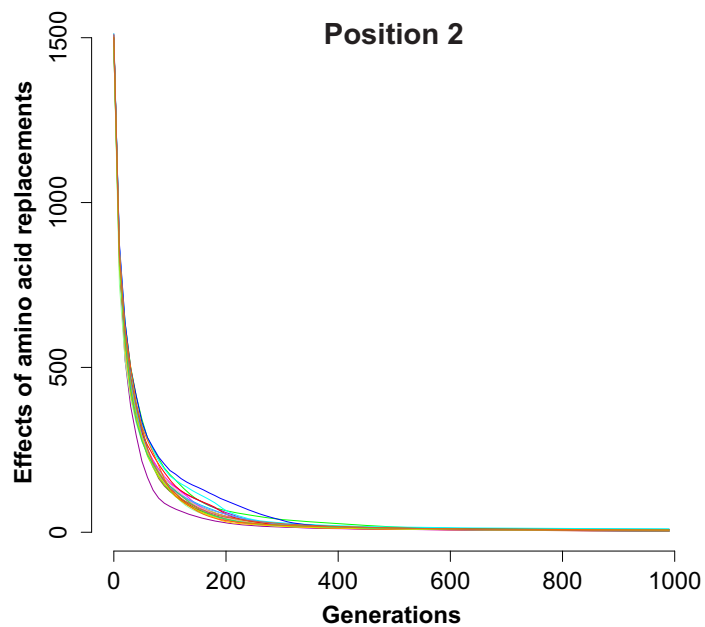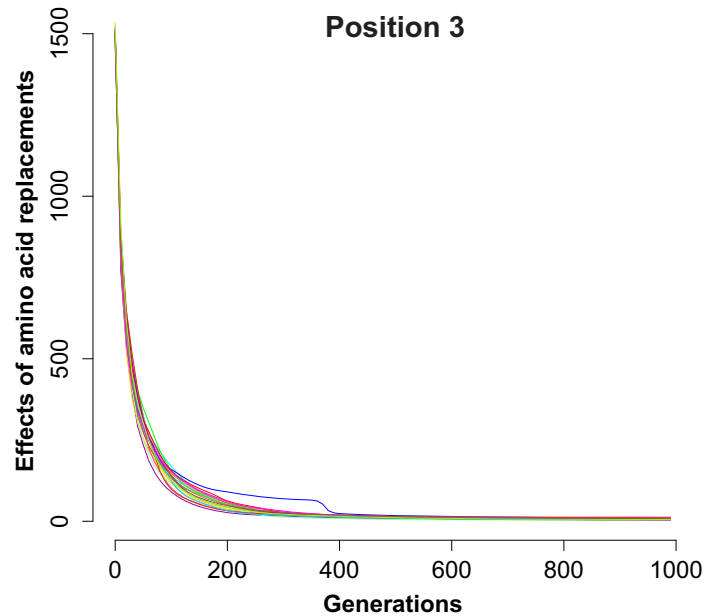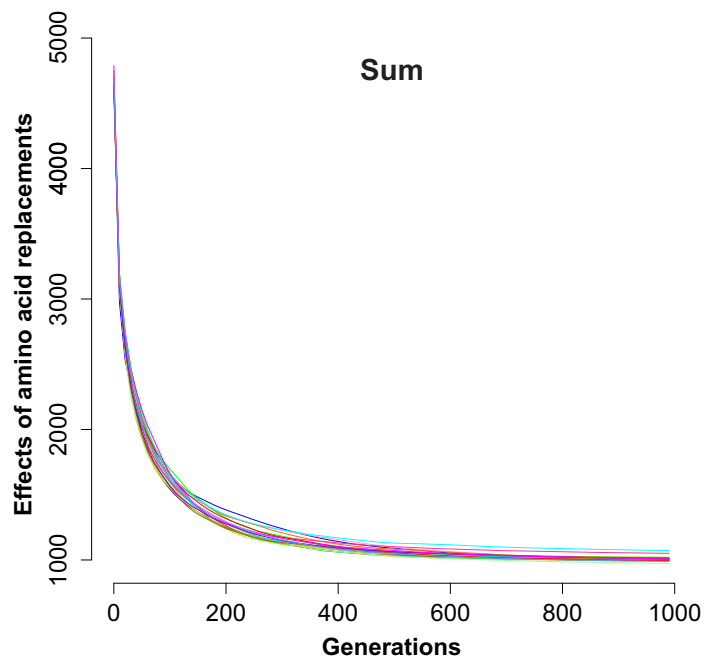

Supplement: S1 Fig — The objective function was minimized for the three codon positions and the sum of costs for the all codon positions. (PDF) [file pone.0201715.s001.pdf]
